# Dynamic changes in lysosome‐related pathways in APP/PS1 mice with aging

**DOI:** 10.1002/mco2.540

**Published:** 2024-04-10

**Authors:** Zhendong Xu, Jichang Hu, Zhen Wei, Yu Lei, Henok Kessete Afewerky, Yang Gao, Lu Wan, Longfei Li, Ling Lei, Yi Liu, Fang Huang, Tong Yu, Jian‐Zhi Wang, Hong‐Lian Li, Rong Liu, Xiaochuan Wang

**Affiliations:** ^1^ Department of Pathophysiology School of Basic Medicine Key Laboratory of Education Ministry/Hubei Province of China for Neurological Disorders Tongji Medical College, Huazhong University of Science and Technology Wuhan China; ^2^ Co‐innovation Center of Neuroregeneration Nantong University Nantong China

**Keywords:** Alzheimer's disease, autophagy–lysosome system, Aβ, endosomal/lysosome system, SGK1/FOXO3a pathway, transcriptome sequencing

## Abstract

Senile plaque, composed of amyloid β protein (Aβ) aggregates, is a critical pathological feature in Alzheimer's disease (AD), leading to cognitive dysfunction. However, how Aβ aggregates exert age‐dependent toxicity and temporal cognitive dysfunction in APP/PS1 mice remains incompletely understood. In this study, we investigated AD pathogenesis and dynamic alterations in lysosomal pathways within the hippocampus of age‐gradient male mice using transcriptome sequencing, molecular biology assays, and histopathological analyses. We observed high levels of β‐amyloid precursor protein (APP) protein expression in the hippocampus at an early stage and age‐dependent Aβ deposition. Transcriptome sequencing revealed the enrichment of differential genes related to the lysosome pathway. Furthermore, the protein expression of ATP6V0d2 and CTSD associated with lysosomal functions exhibited dynamic changes with age, increasing in the early stage and decreasing later. Similar age‐dependent patterns were observed for the endosome function, autophagy pathway, and SGK1/FOXO3a pathway. Nissl and Golgi staining in the hippocampal region showed age‐dependent neuronal loss and synaptic damage, respectively. These findings clearly define the age‐gradient changes in the autophagy–lysosome system, the endosome/lysosome system, and the SGK1/FOXO3a pathway in the hippocampus of APP/PS1 mice, providing new perspectives and clues for understanding the possible mechanisms of AD, especially the transition from compensatory to decompensated state.

## Graphical abstract

During the compensation period, ATP6V0d2 protein was significantly elevated in the hippocampus of APP/PS1 mice, which increased the fusion of autophagosomes and endosomes with lysosomes, thus enhancing the degradation of APP protein. As a result, the modest generation of Aβ could promote the activation of SGK1, thereby exerting its anti‐inflammatory, neuroprotective, and resistance to Aβ toxicity. In the decompensation stage, the significant decrease of ATP6V0d2 led to the blockage of the fusion of autophagosome–lysosome and endosome–lysosome, and a large amount of APP was deposited into the early endosome and autophagosome, which was sheared by BACE1 to produce a large amount of Aβ, thus inhibiting the expression of SGK1. SGK1 deficiency induced a decreased phosphorylation of FOXO3a, resulting in increased nucleation of nonphosphorylated FOXO3a and elevated expression of apoptosis genes and lysosome genes. Further deterioration of Aβ pathology resulted in significant loss of neurons and synaptic damage in the hippocampus of aged APP/PS1 mice.

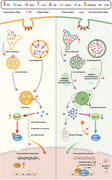

## 1 BACKGROUND

Alzheimer's disease (AD) is a neurodegenerative disease of multifactorial etiology characterized by chronic and progressive cognitive decline. Senile plaques, composed primarily of amyloid β (Aβ) peptide aggregations, and neurofibrillary tangles, formed by abnormally hyperphosphorylated tau (p‐tau) protein, are two major neuropathological changes seen in patients with AD. Tau and Aβ are mutually causal and promote each other, thereby accelerating AD progression. Abnormal Aβ metabolism is among the earliest manifestations observed in the brains of AD patients. The Aβ cascade hypothesis on the pathogenesis of AD proposes that abnormal Aβ protein accumulation is the initiating factor of the disease, subsequently leading to neuronal degeneration and dysfunction resulting in cognitive decline and mental behavior disorders, leading eventually to death.^1^, ^2^, ^3^ Despite the large number of relevant studies on the pathology of AD, the specific mechanism involved in abnormal Aβ protein metabolism and the associated toxic effects that lead to AD remain to be clarified.

Autophagy is a lysosomal‐dependent process that serves to recycle energy through the degradation of organelles and abnormal proteins, thereby maintaining cellular homeostasis. In the brains of AD patients, autophagy dysfunction can markedly affect metabolism and is considered to underlie the harmful accumulation of Aβ protein in the brain of AD patients.^4^ An age‐dependent increase in Aβ and p‐tau contents can result in a reduction in the levels of functional proteins in autophagosomes and mitochondria, leading to defective autophagy and mitochondrial dysfunction.^5^ Aβ can also negatively affect lysosomal function, resulting in impaired autophagy and severe cell damage.^6^ It has been reported that the autophagy activator rapamycin can exert beneficial effects in early‐stage AD but is harmful as a late‐stage intervention.^7^ These studies indicated that autophagy flux changes dynamically during the progression of AD. Aβ is generated via the sequential cleavage of β‐amyloid precursor protein (APP), a process that occurs primarily in the endosome following APP endocytosis.^8^ Under the acidic conditions of the endosome, APP is first cleaved by BACE1, generating the APPβ and C99 fragments, the latter of which is further cleaved by γ‐secretase in the endosomal membrane, yielding the Aβ peptide.^9^ Endosomal dysfunction is also observed in neurons of patients with early‐stage AD,^8^ Additionally, an electron microscopy‐based study found that the number of abnormal endosomes was increased in triploid APP transgenic mice, a commonly used animal model of AD,^10^ This finding further emphasizes that abnormal APP metabolism resulting from endosomal dysfunction may be involved in the pathogenesis of this disease.

Serum and glucocorticoid‐regulated kinase 1 (SGK1) plays a crucial role in cellular stress responses, which is involved in the regulation of multiple processes, including hormone release, neuronal excitability, inflammation, transport regulation, coagulation, cell proliferation, and apoptosis.^11^ Additionally, SGK1 may be involved in the pathophysiology of several brain diseases, such as Parkinson's disease, schizophrenia, depression, and AD, and may also play a significant role in the regulation of neuronal function.^12^ One study reported that the expression of SGK1 is decreased in the brain of AD patients and that SGK1 can promote nonamyloidogenic APP processing and Aβ degradation as well as upregulate the expression of synaptic plasticity‐related proteins, playing a notable role in the treatment of AD memory disorder.^13^ In vitro, SGK1 protects neurons from injury by phosphorylating forkhead box O3a (FOXO3a), resulting in reduced transcriptional activity and anti‐apoptotic effects.^14^ Increased hippocampal FOXO3a abundance was reported to be positively correlated with amyloid plaque density, Aβ42 content, and clinical dementia score in AD.^15^ Furthermore, the SGK1/FOXO3a pathway can influence the expression of lysosome‐related genes,^16^ thereby potentially influencing the functionality of the autophagy–lysosome system and the endosomal/lysosome system.^17^ However, the specific impact of the SGK1/FOXO3a pathway on the autophagy–lysosome system and endosomal/lysosome system remains unclear but is likely to involve a unique regulatory mechanism.

To address this knowledge gap, in this study, we explored the dynamic changes occurring in Aβ metabolism in the process of AD using a holistic approach. First, we used transcriptome sequencing to identify relevant molecular markers in the hippocampus of APP/PS1 mice of different age ranges that would allow the determination of the changes in the Aβ metabolic pathway during AD. Subsequent bioinformatic, biochemical, morphological, and behavioral analysis showed that, in APP/PS1 mice, the autophagy–lysosome system, the endosome/lysosome system, and the SGK1/FOXO3a pathway underwent compensatory‐to‐decompensatory pathophysiological alterations with increasing age. These findings suggested that Aβ metabolism is involved in the course of AD and shows dynamic changes in the compensatory, plateau, and decompensatory stages of the disease, providing new clues for elucidating the pathogenesis of this neurodegenerative disorder.

## 2 RESULTS

### 2.1 APP/PS1 mice show age‐dependent cognitive dysfunction and accumulation of brain β‐amyloid plaques

**FIGURE 1:**
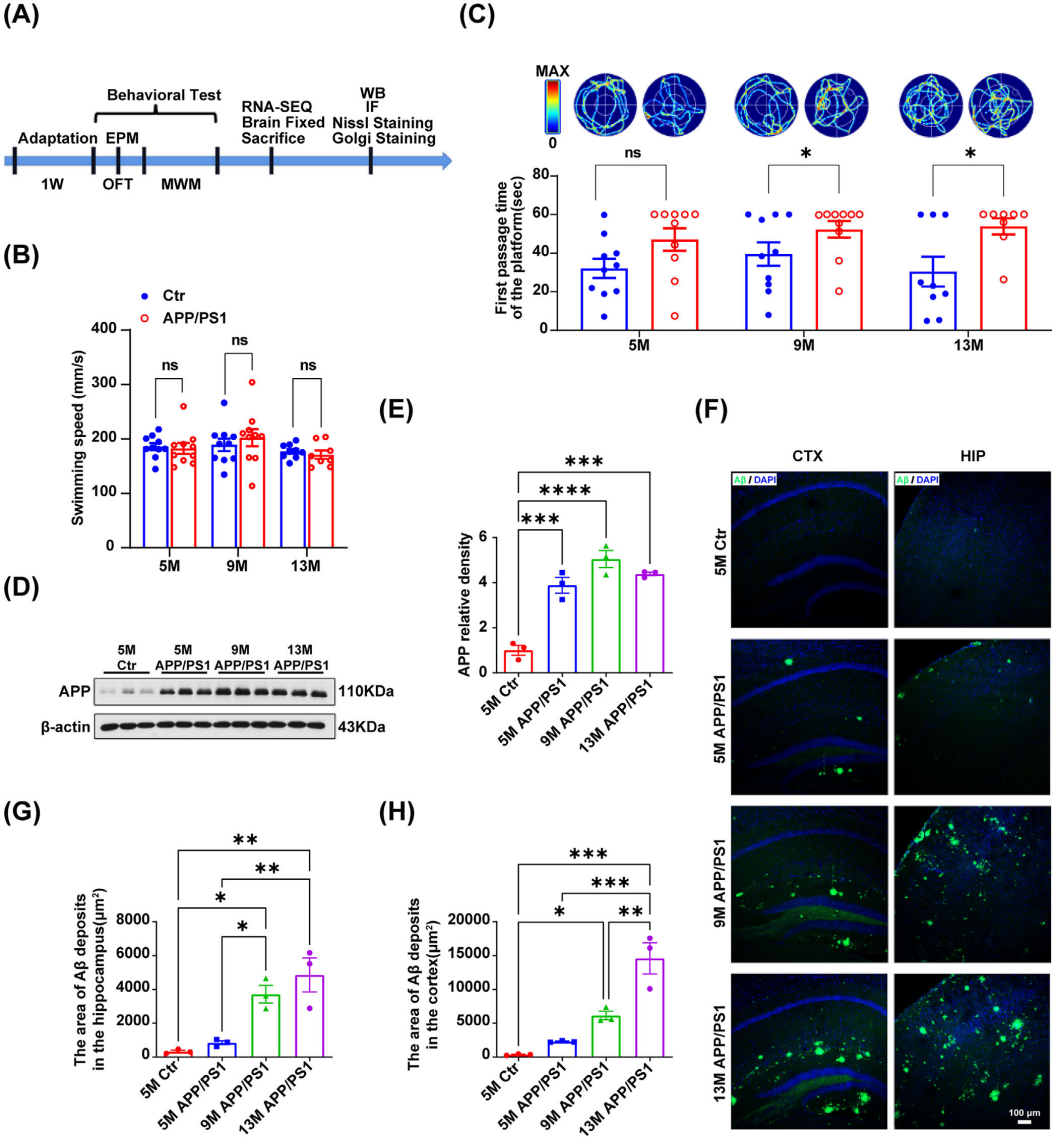
Age‐dependent cognitive impairment and accumulation of brain β‐amyloid plaques in APP/PS1 mice. APP/PS1 mice aged 5 months, 9 months and 13 months were divided into three groups according to age gradient, and c57 wild‐type mice aged 5 months, 9 months and 13 months were used as controls, and then behavioral tests were conducted. (A) Diagram of the experiment. (B–C) The Morris water maze (MWM) test was performed to evaluate the spatial memory of mice. (B) The mean swimming speed of each group. Data are presented as mean ± SEM (5 M Ctr, *n* = 10; 5 M APP/PS1, *n* = 10; 9 M Ctr, *n* = 10; 9 M APP/PS1, *n* = 10; 13 M Ctr, *n* = 9; 13 M APP/PS1, *n* = 8, *p < 0.05). (C) The first passage time of the platform in the target quadrant within 1 min after removing the platform and the representative trajectory of mice in the probe trial. (D) Levels of APP in the hippocampus were detected by Western blotting analysis, and ACTB was used as a loading control. (E) Quantitative analysis of the blots. (F) Representative immunofluorescence staining images showing amyloid‐β (Aβ) deposits in the hippocampus and cortex of wild‐type (Ctr) and APP/PS1 mice. Quantification of Aβ deposits in the hippocampus (G) and cortex (H) of Ctr and APP/PS1 mice by Image‐Pro Plus 6.0 software. Data are presented as mean ± SEM (*n* = 3 per group. *p < 0.05, **p < 0.01, ***p < 0.001, ****p < 0.0001). Aβ, amyloid β protein; EPM, elevated plus maze; MWM, Morris water maze; OFT, open‐field test; WB, Western blotting.

First, we investigated whether APP/PS1 mice, a classic animal model of AD with Aβ toxicity, exhibit age‐dependent cognitive dysfunction. For this, APP/PS1 mice were divided into three groups according to age (5, 9, and 13 months), with age‐matched c57 wild‐type (WT) mice serving as controls. Experiments were performed after 1 week of acclimatization in the animal room (Figure 1A). To explore the relationship between memory dysfunction and age, the mice were subjected to the Morris water maze (MWM). The results indicated a trend of increased latency to find the hidden platform in APP/PS1 mice in all age groups compared to age‐matched WT mice during the five consecutive days of the training period (Figure S1A‐C). However, no significant difference was detected in swimming speed among the six groups (Figure 1B), indicating that APP/PS1 mice may have decreased spatial learning ability, but their motor ability is unaffected. The probe trial was conducted on day 6 of the MWM test. For this, the hidden platform was removed, and the mice were allowed to freely explore the water maze for 60 s. The results showed that compared with their WT counterparts, APP/PS1 mice at the ages of 9 and 13 months took significantly longer to cross the original platform location for the first time and spent less time in the target quadrant. Five‐month‐old APP/PS1 mice also took longer to reach the original platform location for the first time, although the difference was not significant (Figure 1C). These findings suggested that APP/PS1 mice begin to develop mild memory impairment as early as 5 months of age and that the extent of cognitive dysfunction is age‐dependent.

To assess whether APP/PS1 mice show age‐dependent anxiety‐like behavior, we first employed the open‐field test and recorded the percentage of time spent in the central area of the test apparatus. We found that compared with 5‐month‐old WT mice, APP/PS1 mice in the three age groups showed a decreasing trend in the percentage of time spent in the central area, although the difference did not reach significance (Figure S1D‐E). Anxiety‐like behavior in APP/PS1 mice was also evaluated using the elevated plus maze (EPM) test. The results showed that both the number of entries into the open arms and the time spent in the open arms by APP/PS1 mice of the three age groups showed a decreasing trend compared with those of 5‐month‐old WT controls; however, the difference was again not significant (Figure S1F‐G). Altogether, the results of the behavioral experiments indicated that APP/PS1 mice may not display age‐dependent anxiety‐like behavior.

Aβ is produced via the amyloidogenic processing of APP. Western blotting was used to detect APP protein levels in APP/PS1 mice of the three age groups. The results showed that the hippocampal APP protein level was increased in 5‐month‐old APP/PS1 mice compared with that in age‐matched WT controls and was further increased at 9 and 13 months of age (Figure 1D,E). However, there was no difference in age‐gradient c57 mice (Figure S2A,B). Next, we used the Aβ peptide‐specific antibody 6E10 to detect Aβ plaque accumulation in the hippocampus and cortex of the mouse brain using immunofluorescence (Figure 1F). Aβ plaque accumulation was observed in 5‐month‐old APP/PS1 mice but was absent in age‐matched WT controls. Moreover, the observed accumulation in the cortex and hippocampus showed significant age dependency, particularly in 13‐month‐old APP/PS1 mice (Figure 1F–H). However, no difference in Aβ plaque accumulation was observed among c57 mice of the three age groups (Figure S2C). These findings indicated that there is a gradual, age‐dependent increase in Aβ plaque accumulation in the APP/PS1 mouse brain.

### 2.2 Transcriptome sequencing results revealed changes in lysosomal pathways in the hippocampus area of age gradient APP/PS1 mice brain

**FIGURE 2:**
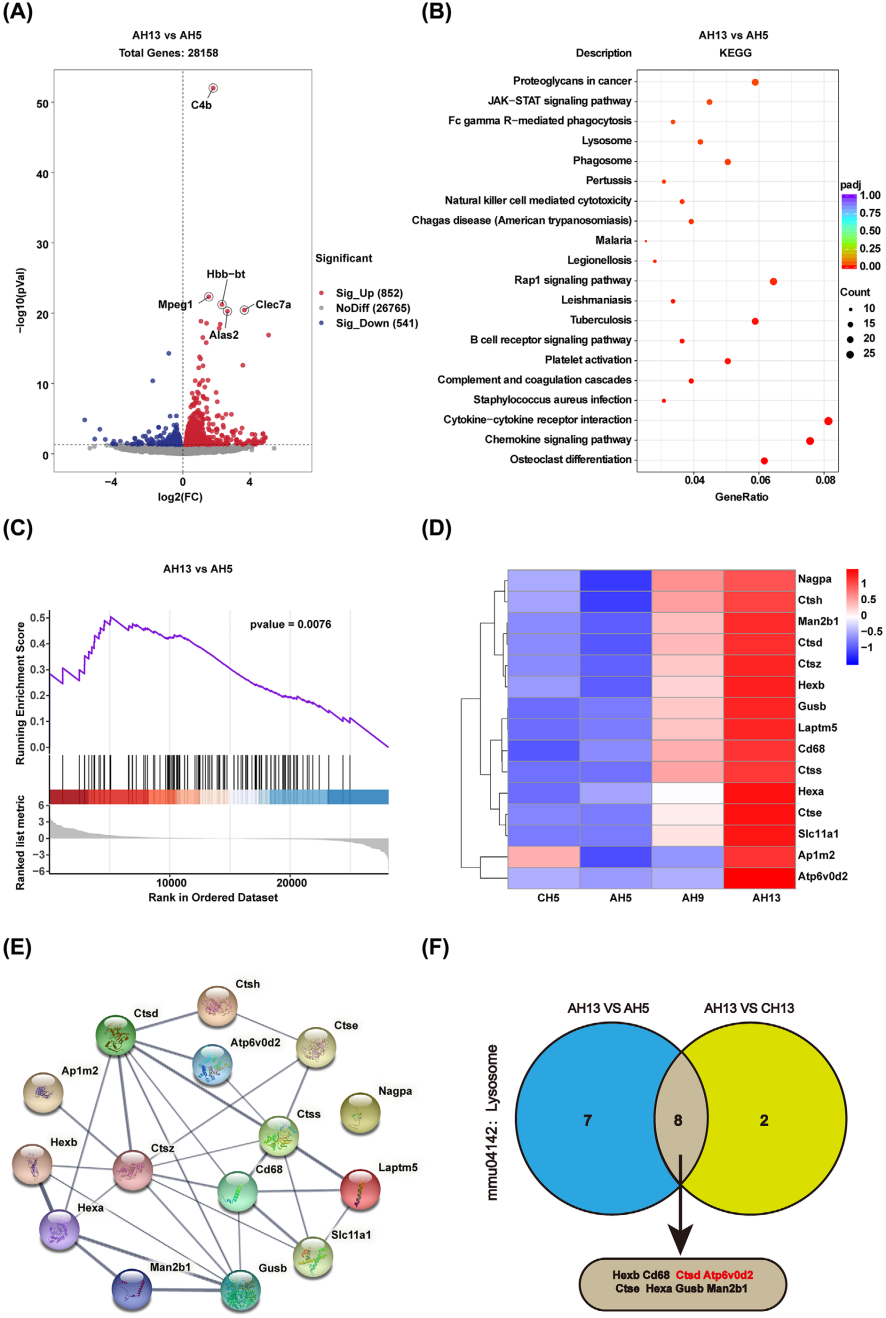
The lysosomal pathway revealed via RNA‐seq data analysis was significantly changed in APP/PS1 mice hippocampus with increased age. (A) The different expression genes of the hippocampus between 5‐month‐old (AH5) and 13‐month‐old (AH13) APP/PS1 mice. (B) Top20 KEGG pathways most enriched in the different gene expressions of the hippocampus between 5‐month‐old (AH5) and 13‐month‐old (AH13) APP/PS1 mice. (C) Lysosome KEGG term of gene set enrichment analysis. (D) The heatmap of differential expression genes in the lysosome pathway of four groups of mice. (E) PPI network of targets extracted from (D). (F) The Venn diagram to find genes shared between 13‐month‐old (AH13) APP/PS1 mice versus 5‐months‐old (AH5) APP/PS1 mice and 13‐month‐old (AH13) APP/PS1 mice versus 13‐month‐old (CH13) WT mice.

**FIGURE 3:**
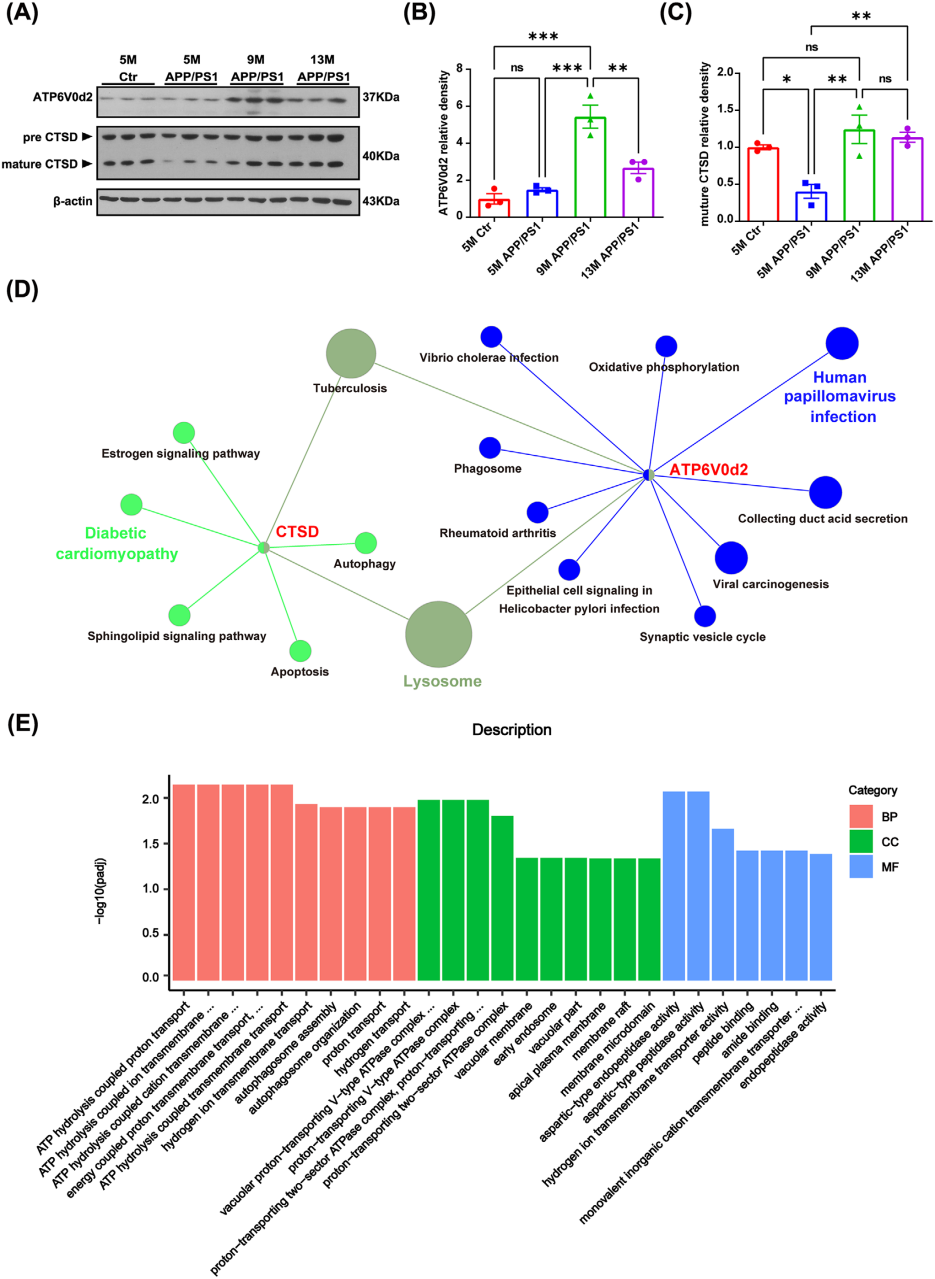
The lysosomal function was dynamically changed in the APP/PS1 mice hippocampus with increased age. (A) Western blotting was performed to detect the expression of ATP6V0d2 and CTSD proteins in the hippocampus. (B‐C) Quantitative analysis of the blots. Data are presented as mean ± SEM (*n* = 3 per group. **p < 0.01, ***p < 0.001). (D) KEGG pathway analysis of the genes (ATP6V0d2 and CTSD) using Clue GO software and the *p*‐value was set at < 0.05. (E) Gene Ontology (GO) analysis of the genes (ATP6V0d2 and CTSD) into three categories: biological process (BP), cellular component (CC), and molecular function (MF).

To further explore the mechanisms underlying the dynamic changes in Aβ aggregation and the related pathophysiologic mechanisms in the brains of APP/PS1 mice of different ages, we undertook a transcriptome sequencing analysis of the hippocampus of the animals. As expected given that AD is mostly age‐dependent,^18^ we found that the differences in gene expression and enriched pathways among the three age groups of APP/PS1 mice differed significantly from those observed among c57 mice of different ages (Figures S2D and S3). Next, differentially expressed genes (DEGs) between 5‐ and 13‐month‐old APP/PS1 mice were identified through analysis of transcriptome sequencing data (Figure 2A) and were submitted to KEGG pathway enrichment analysis. The top 20 enriched KEGG pathways included osteoclast differentiation, chemokine signaling, cytokine‐cytokine receptor interaction, complement cooperative cascade, platelet activation, B cell receptor signaling, and natural killer cell‐mediated cytotoxicity. The KEGG pathways identified also included phagosome, lysosome, and Fc gamma‐R‐mediated phagocytosis pathways (Figure 2B). In AD, abnormal protein aggregation leads to impaired protein degradation, and Aβ aggregation occurs in the brains of APP/PS1 mice. Thus, we focused on the lysosomal pathway, given its potential association with AD pathology, and that significant changes in this pathway were identified through KEGG analysis. Most of the DEGs in the lysosome pathway were upregulated based on gene set enrichment analysis (GSEA) (Figure 2C). Accordingly, we next generated a heatmap of DEGs related to the lysosome pathway based on RNA‐seq data for hippocampal samples from 5‐month‐old WT mice and APP/PS1 mice from the three age groups (Figure 2D). We found that the products of these genes formed a complex protein–protein interaction (PPI) network comprising 15 nodes and 30 edges, with an average node degree of 4 (Figure 2E). This demonstrated that the genes encoding these lysosome‐related degradationenzymes, such as Cathepsin D (CTSD), Cathepsin Z (CTSZ), and Cathepsin S (CTSS), were closely linked with other genes in the PPI network, suggesting that impaired activity of these enzymes may play a key role in lysosomal dysfunction in APP/PS1 mice. Next, to elucidate the mechanism underlying the significant increase in the expression of lysosome‐associated genes in aged APP/PS1 mice, a Venn diagram was generated to identify DEGs shared between 13‐month‐old and 5‐month‐old A PP/PS1 mice and between 13‐month‐old APP/PS1 mice and 13‐month‐old WT mice (Figure 2F). We analyzed the expression of genes, namely, Atp6v0d2 and Ctsd, which may affect lysosomal function and the occurrence of AD pathology (for details, see Figure 3). Meanwhile, we also found the mRNA expression levels of ATP6V0d2 (hippocampus GSE36980) and CTSD (hippocampus GSE29378) according to the AlzData database (http://www.alzdata.org/) were significantly increased, which was consistent with the results of transcriptome sequencing and verified in the human hippocampus (Figure S4). In summary, our findings suggested that lysosomal physiology undergoes significant changes in the hippocampus of APP/PS1 mice in an age‐dependent manner and that the ATP6V0d2 and CTSD genes may play an important role in lysosomal dysfunction and the occurrence of AD pathology.

### 2.3 Lysosome function undergoes dynamic changes in the hippocampus of APP/PS1 mice in an age‐dependent manner

To better understand the dynamic age‐related changes in lysosome function occurring in the hippocampus of APP/PS1 mice, we measured the protein expression levels of ATP6V0d2 and CTSD by immunoblotting (Figures 3A and S5A). The results showed that hippocampal ATP6V0d2 protein expression was significantly higher in 9‐month‐old APP/PS1 mice than in both 5‐month‐old and 13‐month‐old APP/PS1 mice, exhibiting a trend of initially rising and subsequently falling with age. However, no difference in hippocampal ATP6V0d2 protein expression was detected between 5‐month‐old APP/PS1 mice and age‐matched WT mice (Figure 3B). Compared with that seen in 5‐month‐old WT mice, APP/PS1 mice of the same age had significantly lower levels of the mature form of CTSD. However, compared with 5‐month‐old APP/PS1 mice, the expression of CTSD was significantly increased in 9‐ and 13‐month‐old APP/PS1 mice. No difference in CTSD expression was observed between 9‐month‐old and 13‐month‐old APP/PS1 mice (Figure 3C) or among c57 mice of the three age groups (Figure S5B‐C). KEGG pathway enrichment analysis showed that the genes encoding products were co‐enriched in tuberculosis and lysosome pathways, while gene ontology (GO) functional analysis indicated that both genes are involved in energy metabolism, proton transport, and protein degradation (Figures 3D,E, and S5D‐F). These results suggested that lysosomal function in the hippocampus of APP/PS1 mice first increased and then decreased with increasing age.

### 2.4 The autophagy–lysosome and endosome/lysosome systems in the hippocampus of APP/PS1 mice showed dynamic age‐related changes

**FIGURE 4:**
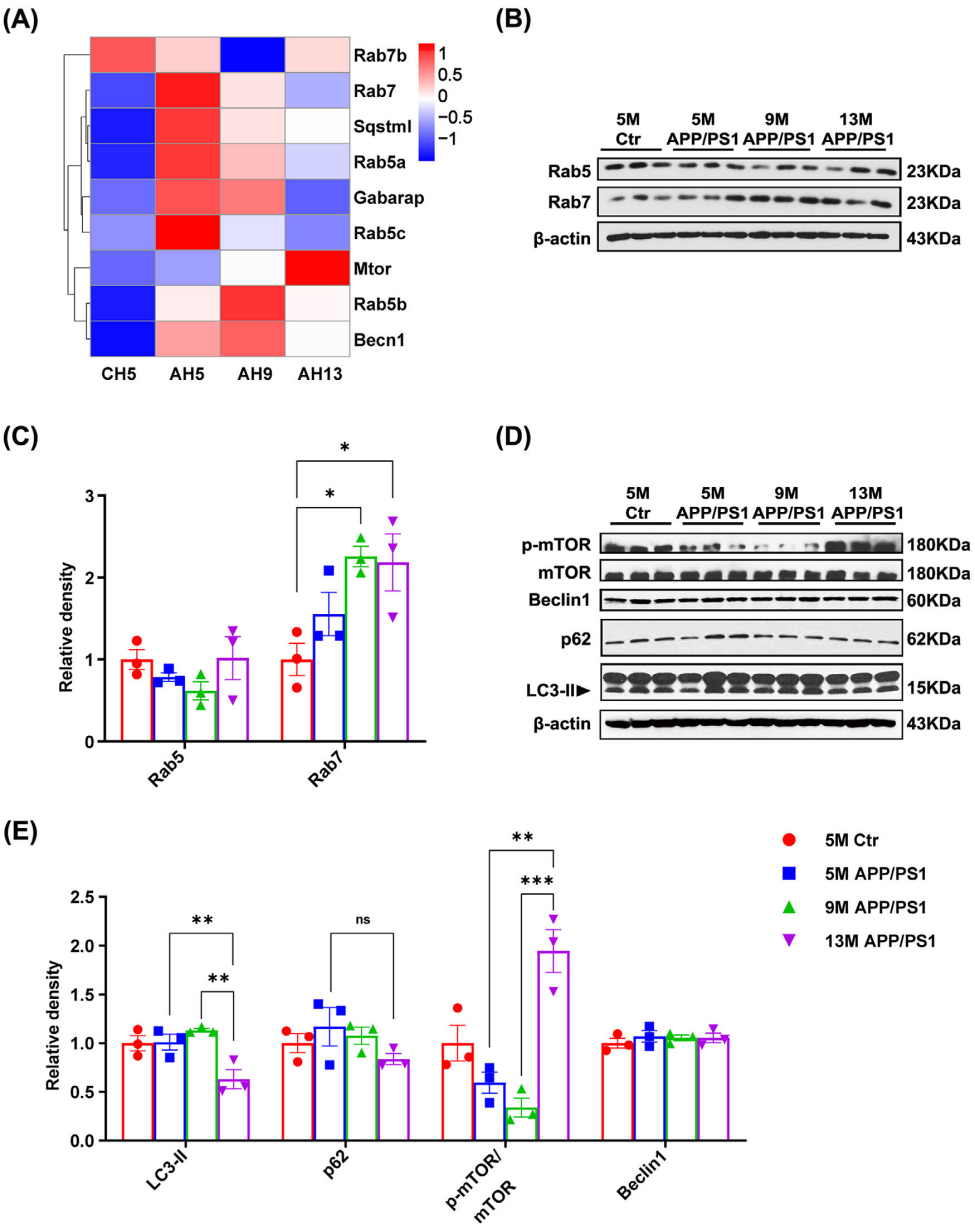
The endosome and autophagy pathways underwent dynamic changes in the APP/PS1 mice hippocampus as age increased. (A) The heatmap of autophagy–lysosome or endosome–lysosome pathway functional genes in the four groups of mice. (B) Western blotting was performed to detect the protein in the endosome–lysosome pathway. (C) Quantitative analysis of the blots for panel (B). (D) Western blotting was performed to detect the protein in the autophagy–lysosome pathway. (E) Quantitative analysis of the blots for panel (D). Data are presented as mean ± SEM (*n* = 3 per group. ns: not significant, *p < 0.05, **p < 0.01, ***p < 0.001).

The lysosome is the principal site of degradation in the autophagy–lysosome and endosome/lysosome systems. Here, we found that both systems undergo dynamic changes during AD progression, as can be seen in the heatmap of DEGs between APP/PS1 and control mice (Figure 4A). Accordingly, we first evaluated the expression of the endosomal marker proteins Rab5 and Rab7 by Western blot (Figure 4B). The results showed that compared with 5‐month‐old WT mice, APP/PS1 mice showed an age‐dependent increase in Rab7 expression, especially in the hippocampus of 9‐month‐old and 13‐month‐old AD model mice (Figure 4C). Compared with young APP/PS1 mice, the expression of Rab5 in the hippocampus of 13‐month‐old APP/PS1 mice showed an upward trend (Figure 4C). However, no difference in Rab5 or Rab7 expression was detected among the three groups of c57 mice (Figure S6A–C).

Western blot analysis of autophagy‐related proteins (Figure 4D) showed that LC3‐II expression exhibited an increasing trend in the hippocampus of APP/PS1 mice at 5 and 9 months of age and was significantly decreased at 13 months of age (Figure 4E). The knockout of the p62 gene can lead to AD‐like lesions.^19^ In this study, we also found that the expression of p62 in APP/PS1 mice showed a decreasing trend with increasing age (Figure 4E). However, no significant difference in p62 expression was observed among the three groups of c57 mice (Figure S6A, S6D). These results suggested that autophagy is enhanced in the early stages of AD and degradation is increased, while the storage of autophagy marker protein p62 decreased in the late stage of the disease due to the consumption of autophagy flux. To explore the molecular mechanism underlying autophagy dynamics in APP/PS1 mice, we measured the levels of mTOR and beclin1, key autophagy‐related proteins. Compared with 5‐month‐old WT mice, the p‐mTOR/total mTOR ratio in the hippocampus of APP/PS1 mice showed a downward trend at the ages of 5 and 9 months, but increased significantly at 13 months of age (Figure 4E); meanwhile, no difference in beclin1 expression was found among the three groups of APP/PS1 mice (Figure 4E) or among the three groups of WT mice (Figure S6A, 6E). These observations suggested that the inhibition of autophagy in the hippocampus of 13‐month‐old APP/PS1 mice was likely the result of mTOR phosphorylation.

### 2.5 The SGK1/FOXO3a pathway underwent dynamic changes related to the survival of hippocampal neurons in APP/PS1 mice

**FIGURE 5:**
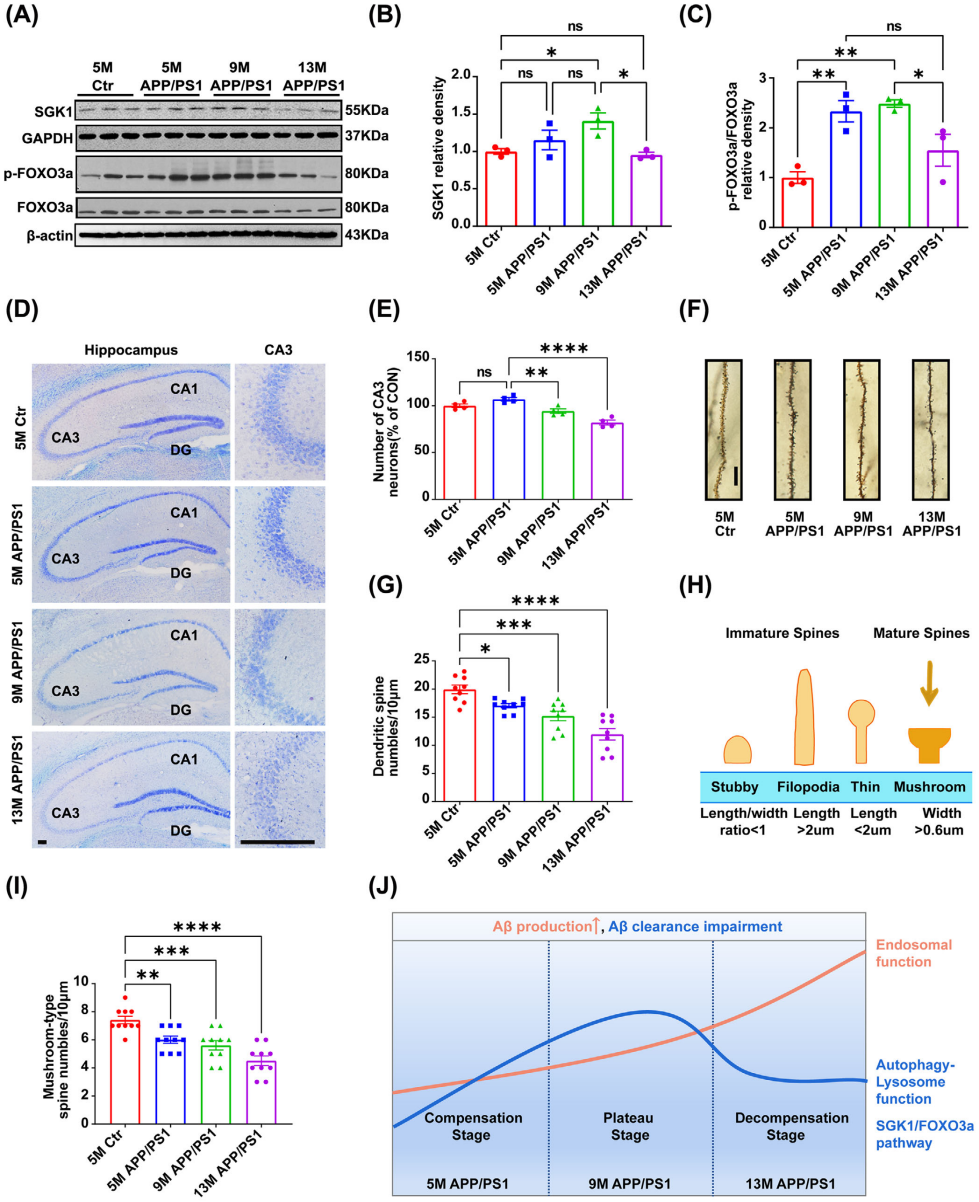
Alteration of the SGK1/FOXO3a pathway in the hippocampus of APP/PS1 mice, which also exhibited an increase in age‐dependent neuron loss and synaptic loss. (A) Western blotting was performed to detect the protein of the SGK1/FOXO3a pathway. (B‐C) Quantitative analysis of the blots about the SGK1/FOXO3a pathway. Data are presented as mean ± SEM (*n* = 3 per group. ns: not significant, *p < 0.05, **p < 0.01). (D) Representative Nissl staining images showing Nissl bodies in the CA3 region of the hippocampus in wild‐type (Ctr) and APP/PS1 mice (bar = 50 µm). (E) Quantifications of Nissl bodies in the CA3 regions of the hippocampus in Ctr and APP/PS1 mice by Image‐Pro Plus 6.0 software. Data are presented as mean ± SEM (*n* = 4 per group. ns: not significant, **p < 0.01, ****p < 0.0001). (F) Representative Golgi staining images (bar = 10 µm). (G) The quantification of spine density by Image J software. (H) Common dendritic spine types are found in the brain. (I) The quantification of mushroom‐type spines. Data are presented as mean ± SEM (*n* = 9 neuron dendrites/group, sampled from 4 mice/group. *p < 0.05, **p < 0.01, ***p < 0.001, ****p < 0.0001). (J) Schematic diagram of the functional trends of the autophagy pathway, lysosomal function, and SGK1/FOXO3a pathway during the pathogenesis of AD in APP/PS1 mice.

SGK1 can phosphorylate FOXO3a, which subsequently translocates from the nucleus, resulting in decreased FOXO3a‐mediated nuclear transcriptional activity and antiapoptotic effects.^14^ Accordingly, we next investigated the putative changes in the SGK1/FOXO3a pathway related to the survival of hippocampal neurons in APP/PS1 mice (Figure S7A). First, we generated a heatmap of DEGs based on SGK1/FOXO3a pathway‐related transcriptomic data (Figure S7B). The products of these genes formed a complex PPI network comprising nine nodes, with an average node degree of 2.44 and 11 edges (Figure S7C). This demonstrated that the Sgk1 gene is closely related to other genes in the PPI network, suggesting that these genes may play a key role in the FOXO3a pathway in APP/PS1 mice. The results further showed that the hippocampal expression of the Sgk1 gene in APP/PS1 mice increased with age (Figure S7B). Next, to elucidate the mechanism involved in the marked increase in the levels of FOXO3a pathway genes in aged APP/PS1 mice, a Venn diagram was used to identify genes shared between 13‐month‐old APP/PS1 mice and 5‐month‐old APP/PS1 mice and between 13‐month‐old APP/PS1 mice and 13‐month‐old WT mice (Figure S7D). We analyzed the genes, namely, Sgk1 and Foxo3a, both of which are known to affect the FOXO and PI3K‐AKT signaling pathways reported to be closely related to AD pathology (Figure S7E). Next, we assessed the expression levels of proteins in the SGK1/FOXO3a pathway in the hippocampus of APP/PS1 mice using Western blotting (Figure 5A). The results showed that compared with 5‐month‐old WT mice, the p‐FOXO3a/total FOXO3a ratio was significantly increased in APP/PS1 mice at the age of 5 months, peaked at the age of 9 months, and decreased at the age of 13 months (Figure 5B). No difference in the p‐FOXO3a/total FOXO3a ratio was seen among the three groups of WT mice (Figure S6A, S6F). The expression of SGK1, an upstream kinase of FOXO3a, also increased between the ages of 5 and 9 months and then decreased sharply at 13 months of age (Figure 5C). These results implied that in APP/PS1 mice, the SGK1/FOXO3a pathway undergoes dynamic changes, which may compromise neuronal survival.

Meanwhile, we also used the SGK1 inhibitor GSK‐650394 to study the effect of SGK1 in the AD pathological process (Figure S8A). After treatment with 5 µM GSK‐650394 in AD model N2a‐APP cells for 48 h, it was found that the p‐FOXO3a/total FOXO3a ratio decreased significantly compared with the DMSO‐treated control group (Figure S8B), suggesting that there may be changes in APP metabolism. Interestingly, we found that the protein levels of APP and sAPPβ were increased in the SGK1 inhibitor‐treated N2a‐APP cells compared with DMSO‐treated N2a‐APP cells (Figure S8C‐D). To further confirm this result, the cleaving enzymes in APP metabolism were detected. The Western blot results showed no significant difference in the protein levels of BACE1 and PS1 between the two groups, whereas the ADAM10 protein levels were decreased in the SGK1 inhibitor‐treated N2a‐APP cells compared with DMSO‐treated N2a‐APP cells (Figure S8E‐G). Besides, the enzyme‐linked immunosorbent assay (ELISA) results showed that the increased levels of Aβ40 and Aβ42 in N2a‐APP cells treated with SGK1 inhibitor compared with DMSO‐treated N2a‐APP cells (Figure S8H‐I), suggesting that SGK1 inhibitor GSK‐650394 treatment affected Aβ production and accumulation. The aggregate findings, therefore, indicated that inhibition of the SGK1/FOXO3a pathway aggravates Aβ pathology in the AD pathological process.

Since the SGK1/FOXO3a pathway is involved in neuronal survival,^20^ we next quantified the number of neurons in the hippocampi of APP/PS1 mice of all age groups using Nissl staining (Figure 5D). Staining results showed that neuronal loss was significantly more extensive in both 9‐month‐old and 13‐month‐old APP/PS1 mice relative to that seen in 5‐month‐old APP/PS1 mice (Figure 5E), consistent with the age‐dependent cognitive dysfunction observed in the AD model mice. These results suggested that age‐dependent neuronal loss occurs in the hippocampus of APP/PS1 mice. Synaptic damage is well known to be closely associated with cognitive decline. Given that the early appearance of soluble Aβ can result in early synaptic dysfunction and that Aβ can accumulate at synapses,^21^ we next investigated the changes in dendritic spine number and morphology in the hippocampus of APP/PS1 mice of various age groups using Golgi staining (Figure 5F). The results showed that compared with 5‐month‐old WT mice, the number of dendritic spines and mushroom‐type spines in the hippocampus of APP/PS1 mice was significantly decreased at 5 months of age and was further decreased at both 9 and 13 months of age (Figure 5G‐I). Dendritic spine damage appeared concomitantly with Aβ accumulation in young APP/PS1 mice, and dendritic spine loss became increasingly critical with increasing age.

Overall, we observed that APP overexpression and abnormal APP metabolism promoted Aβ production and senile plaque formation. We further found that changes in the autophagy–lysosome system, the endosome/lysosome system, and the SGK1/FOXO3a pathway represent compensatory‐to‐decompensatory pathophysiological processes in the AD process. In particular, lysosomal activity was identified as playing a critical role in Aβ clearance in APP/PS1 mice. These phenomena manifested as dynamic changes in Aβ metabolism in the compensatory, plateau, and decompensatory stages of AD in the hippocampus of APP/PS1 mice (Figure 5J).

## 3 DISCUSSION

Compensatory mechanisms are a fundamental physiological phenomenon in living organisms. Under pathological conditions, these mechanisms are compromised, which often results in vicious cycles that promote the continuous aggravation of a pathological state. In this study, we used APP/PS1 mice of different ages, thus mimicking the age‐related pathogenesis of AD, and evaluated Aβ metabolism, especially the dynamic changes in its degradation in the various age groups. We further investigated age‐related behavior in APP/PS1 mice using the open field, EPM (anxiety‐like behavior), and MWM (cognitive impairment) tests. We found that mice exhibited mild, age‐dependent anxiety‐like behavior, consistent with a previous report,^22^ while cognitive impairment, although mild, could already be detected at the age of 5 months, and significantly worsened in an age‐dependent manner. These observations indicated that our APP/PS1 mice well mimicked the dementia manifestations of AD.

APP is a precursor molecule of the Aβ peptide. We found that APP protein was highly expressed in the brains of APP/PS1 mice at an early stage, which may explain the mild cognitive impairment seen in the young APP/PS1 mice during the early stage of AD in the absence of substantial Aβ plaque accumulation. These findings imply that some compensatory mechanism blocks any significant increase of Aβ levels and plaque formation in the early stage; in contrast, the Aβ level increases significantly in the late stage of AD due to the existence of a decompensatory mechanism, which promotes the further increase in the number of Aβ toxicity‐exacerbating plaques, finally triggering dementia that is characteristic of AD. We also undertook a transcriptome sequencing analysis of APP/PS1 mice in the three age groups. The top 20 KEGG pathways associated with the identified DEGs were mainly involved in immune and degradation systems. It has been reported that the occurrence of AD is closely related to immune system imbalance.^23^ Interestingly, we also observed that the number of DEGs in the lysosomal pathway increased markedly with age, suggesting that ATP6V0d2 and CTSD gene dysfunction may negatively affect the degradation systems and may be linked to AD development.

ATP6V0d2 is a key subunit of the lysosomal proton pump, and its functions may be closely related to lysosomal acidification and autophagosome/lysosome fusion, among other crucial physiological roles.^24^ We found that the expression of ATP6V0d2 in the brains of APP/PS1 mice first increased and then decreased with increasing age. The significant rise in ATP6V0d2 expression in the hippocampus of 9‐month‐old APP/PS1 mice may have been due to the enhanced lysosomal activity, which favors Aβ degradation. The hippocampal levels of ATP6V0d2 were significantly downregulated in 13‐month‐old APP/PS1 mice, likely following late decompensation by the lysosome pathway, which resulted in weaker lysosome acidification and impaired autophagosome/lysosome fusion.

CTSD is an enzyme responsible for protein degradation in lysosomes. Under acidic conditions, the precursor of CTSD in lysosomes can be cleaved into its mature form, which has protein‐degrading activity. CTSD is reported to have a degradative effect on Aβ as well as serve as an indicator of lysosome function.^25^ We found that compared with 5‐month‐old WT mice, the level of mature CTSD protein in the hippocampus of 5‐month‐old APP/PS1 mice was significantly decreased, which may have been due to the low basal level of lysosomal acidification in the APP/PS1 mouse hippocampus,^26^ leading to reduced production of mature CTSD. However, the expression of CTSD in the hippocampus in APP/PS1 mice at 9 and 13 months of age was significantly increased, which may have been a compensatory effect of the lysosomal pathway in clearing abnormal proteins. No difference in CTSD expression was observed between 9‐month‐old and 13‐month‐old APP/PS1 mice, possibly due to the sharp decline in ATP6V0d2 protein levels observed in 13‐month‐old APP/PS1 transgenic animals. Therefore, lysosomal acidification disorder does not affect increasing the expression of mature CTSD protein. Meanwhile, owing to the lack of an appropriately acidic environment, lysosomes may be less capable of degrading abnormal proteins. Hippocampal ATP6V0d2 and CTSD gene expression levels were significantly higher, whereas the protein expression level of ATP6V0d2 was lower in 13‐month‐old than in 9‐month‐old APP/PS1 mice; no difference in the levels of mature CTSD was detected between these two groups. Combined, these observations further support the possible existence of late lysosomal degradation decompensation.

Given that the lysosome is the predominant site of degradation for the autophagic and endosomal pathways, we evaluated the expression of the endosomal marker proteins Rab5 and Rab7. We found that the expression of Rab5 showed an upward trend in the hippocampus of APP/PS1 mice at 13 months of age. Meanwhile, compared with WT controls, the protein levels of Rab7 exhibited an age‐dependent increase in APP/PS1 mice. Early APP production in APP/PS1 mice leads to increased endosomal activity, whereby APP is cleaved by BACE1, yielding Aβ.^27^ Meanwhile, endosome–lysosome fusion is enhanced, resulting in the timely removal of Aβ peptides. In the hippocampus of 13‐month‐old APP/PS1 mice, lysosome acidification and fusion are impaired,^17^, ^28^ implying that a large number of late endosomes accumulate in the cytoplasm, representing a key site for massive Aβ production. These pathophysiological events may explain Aβ plaque accumulation in the hippocampus of aged APP/PS1 mice.

SGK1, a protein crucial for neuron survival, plays a role in the nonamyloidogenic APP processing pathway,^13^ facilitating Aβ degradation and enhancing the expression of proteins associated with synaptic plasticity.^29^ SGK1 can also phosphorylate FOXO3a, leading to its export from the nucleus and the subsequent reduction in FOXO3a‐mediated transcriptional activity in this compartment.^30^ Studies have found that the upregulation of FOXO3a activity in the hippocampus is positively correlated with Aβ42 content.^15^ In addition, the SGK1/FOXO3a pathway can influence the expression of lysosomal‐related genes.^16^ Our RNA‐seq results showed that the expression level of the Sgk1 gene in the hippocampus of APP/PS1 mice increases with age. Additionally, we found that the SGK1/FOXO3a pathway is initially activated, but is subsequently inhibited with increasing age in the hippocampus of APP/PS1 mice. The SGK1‐mediated exit of FOXO3a from the nucleus blocks FOXO3a from inducing cell cycle arrest and apoptosis, thereby promoting cell survival.^30^ The export of FOXO3a from the nucleus can also lead to a reduction in the expression of the Rhodopsin‐associated protein kinase 1 (ROCK1) gene and the promotion of the activity of the nonamyloidogenic APP processing pathway, which is beneficial for reducing Aβ accumulation in AD.^31^ This observation suggests that the early (at 5 and 9 months of age) increase in FOXO3a phosphorylation in the hippocampus of APP/PS1 mice may be a compensatory, cell survival‐promoting response to the increased activity of the amyloidogenic pathway. The reduction in phosphorylated FOXO3a content in the hippocampus in 13‐month‐old APP/PS1 mice likely represents decompensatory alterations in the amyloidogenic pathway in the late stage of AD, whereby the entry of nonphosphorylated FOXO3a into the nucleus induces cell cycle arrest and apoptosis, resulting in the formation of numerous Aβ plaques and late neuronal apoptosis. The entry of nonphosphorylated FOXO3a into the nucleus may also promote the transcription of lysosome‐related genes compensatorily, which may explain the rapid increase in the expression of lysosome‐related genes in late AD; however, this possibility requires further verification. It has also been reported that the inhibition of the SGK1/FOXO3a pathway can lead to autophagy dysfunction,^32^ which may explain the significant decrease in LC3‐II contents in the hippocampus of APP/PS1 mice at 13 months of age. FOXO3a‐induced proteolysis further leads to the degradation of the lysosomal proton pump and, consequently, impaired lysosome acidification.^33^ Studies have also shown that FOXO3a is associated with Aβ‐induced neurotoxicity.^34^ Activated FOXO3a translocates to the nucleus and binds to the Bim promoter, thereby mediating neuronal apoptosis.^35^ Cognitive dysfunction in AD is primarily due to neuronal loss. Here, we found that APP/PS1 mice exhibited neuronal loss that was both progressive and age‐dependent. Additionally, the loss of synapses has been reported to be closely associated with cognitive decline. Our results indicated that APP/PS1 mice display progressively more extensive synaptic damage in an age‐dependent manner. The above results also indicate the pathogenesis of AD from the pathological observation.

## 4 CONCLUSION

**FIGURE 6:**
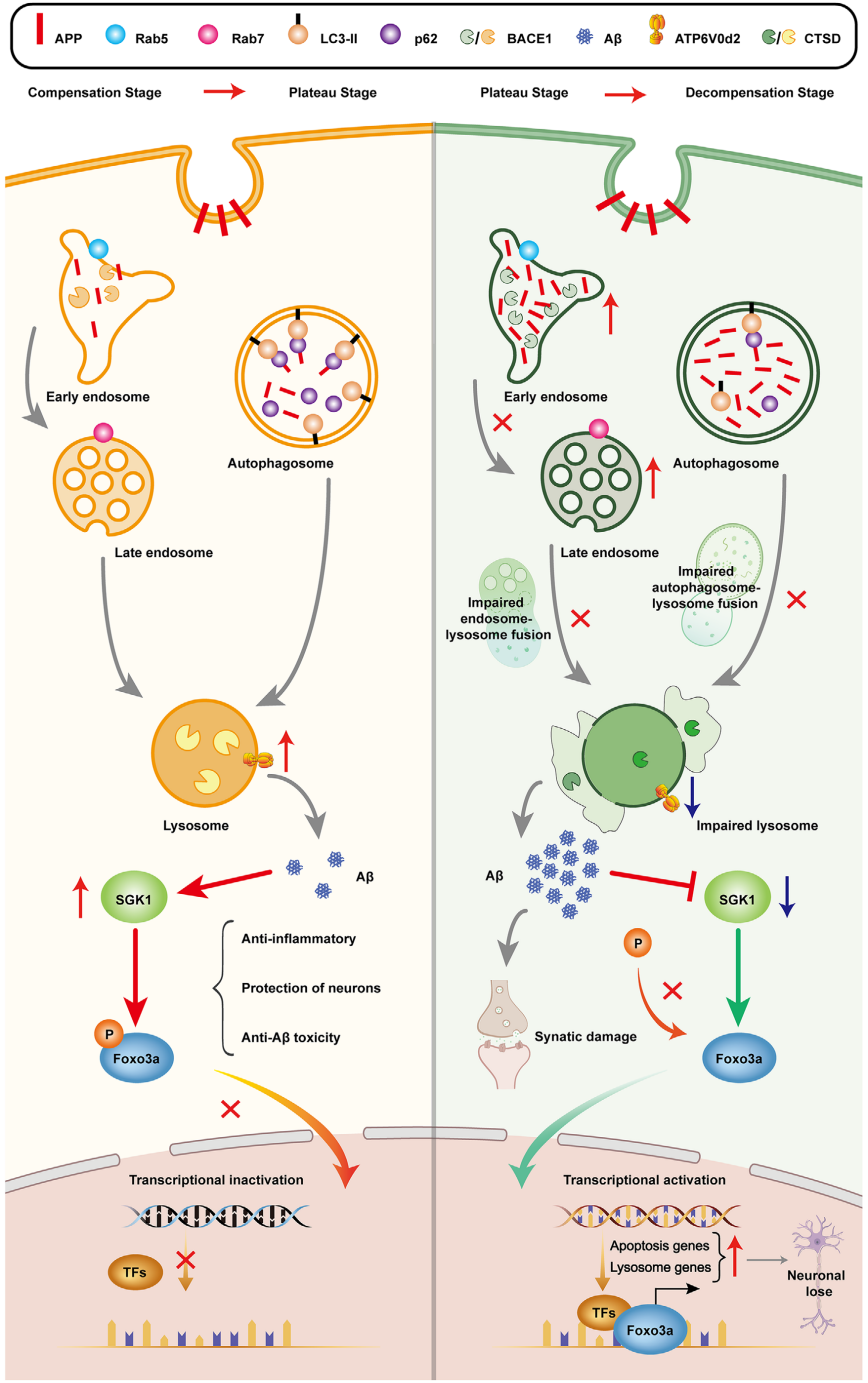
Schematic diagram of molecular mechanisms of the pathologic process from compensatory to decompensated in the hippocampus of age‐gradient APP/PS1 mice. During the compensation period, the ATP6V0d2 protein was significantly elevated in the hippocampus of APP/PS1 mice, which increased the fusion of autophagosomes and endosomes with lysosomes, thus enhancing the degradation of APP protein. As a result, the modest generation of Aβ could promote the activation of SGK1, thereby exerting its anti‐inflammatory, neuroprotective, and resistance to Aβ toxicity. In the decompensation stage, the significant decrease of ATP6V0d2 led to the blockage of the fusion of autophagosome–lysosome and endosome–lysosome, and a large amount of APP was deposited into the early endosome and autophagosome, which was sheared by BACE1 to produce a large amount of Aβ, thus inhibiting the expression of SGK1. SGK1 deficiency induced a decreased phosphorylation of FOXO3a, resulting in increased nucleation of nonphosphorylated FOXO3a and elevated expression of apoptosis genes and lysosome genes. Further deterioration of Aβ pathology resulted in significant loss of neurons and synaptic damage in the hippocampus of aged APP/PS1 mice.

In summary, we analyzed various pathophysiological mechanisms associated with AD pathogenesis in APP/PS1 mice from a dynamic perspective. During the compensation period, elevated ATP6V0d2 in the hippocampus of APP/PS1 mice enhanced the fusion of autophagosomes and endosomes with lysosomes, facilitating APP protein degradation. This led to moderate Aβ production, activating SGK1 for anti‐inflammatory and neuroprotective effects. In the decompensation period, reduced ATP6V0d2 inhibited fusion processes, causing APP accumulation and increased Aβ generation via BACE1. Inhibition of SGK1caused by Aβ overload resulted in FOXO3a dephosphorylation, triggering apoptosis and lysosomal gene upregulation. This progression culminated in severe neuronal loss and synaptic damage in the hippocampus of aged APP/PS1 mice (Figure 6). Our study provides novel ideas for further elucidating the pathogenicity of Aβ toxicity as well as identifying novel therapeutic targets for the prevention of AD.

## 5 MATERIALS AND METHODS

### 5.1 Reagents

Nissl staining solution was sourced from the Beyotime Institute of Biotechnology (C0117; China). The antibodies used in this study are listed in Table S1.

### 5.2 Animals

Male APP/PS1 double‐transgenic mice (strain name: B6C3‐Tg [APPswe, PSEN1dE9] 85Dbo/J)^36^ were purchased from Nanjing Biomedical Research Institute of Nanjing University, and male WT (c57) mice were obtained from the Experimental Animal Center of Tongji Medical College, Huazhong University of Science and Technology. All mice were housed in an air‐conditioned room at a temperature of 23 ± 1°C under a 12‐h/12‐h light‐dark cycle and had free access to food and water. The mice were divided into six groups, 10 mice/group. Male APP/PS1 mice were divided into three age groups (5, 9, and 13 months) for the experiments, with age‐matched WT mice serving as controls. Mice were taken to the behavioral testing rooms at least 1 day before the tests and were euthanized after the final behavioral test. In our experiment, the mice were euthanized and their brains were perfused with normal saline. The hippocampus of three mice was collected for Western blot analysis. The brains of the other three mice were removed after euthanasia, and the hippocampal tissue was divided into two portions for immunofluorescence assay and transcriptome sequencing, respectively. The remaining four mice were also divided into left and right hemispheres for Golgi staining and Nissl staining.

### 5.3 Open‐field test

The open‐field test was used to assess anxiety‐like behavior in the mice. Mice were habituated to the test chamber for 1 h before the test. For testing, the animals were placed in a square box (60 × 60 × 50 cm) with their heads facing the wall, always in the same direction, and were allowed to freely explore for 5 min. The total distance moved and the time spent in the central area were recorded with a tracking camera installed over the test platform. The platform was cleaned after each test.

### 5.4 Elevated plus maze test

The EPM test is a standard method for evaluating anxiety‐related behaviors. Mice were habituated in the test chamber for 1 h before the test. Each mouse was placed at the center of a raised platform shaped like a plus sign, 50 cm above the floor, and illuminated by a 60 W bulb from above. A video camera recorded their movements, tracking them at 5‐min intervals to measure the distance traveled, open and closed arm entries, and time spent. After the test, the maze was cleaned with 75% ethanol. Data from the EPM test were analyzed and presented as percentages.

### 5.5 Morris water maze test

The mice were familiarized with the MWM testing chamber for 1 day. The maze was colored white with harmless flour to aid in tracking mouse movement. The water temperature was maintained at around 25°C. Geometric patterns on the tank's curtain helped mice navigate spatial positions. A platform was placed in one quadrant of the pool, submerged 2 cm below the water surface. The acquisition phase spanned 5 days, with mice trained three times daily in different quadrants. Mice had 60 s to locate the hidden platform; if unsuccessful, they were guided to it and allowed to remain for 20 s. The latency to find the platform was recorded digitally. On day 6, a probe trial was conducted with the platform removed, allowing mice to swim freely for 60 s. Exploration time in the target quadrant and latency to cross the original platform location were recorded.

### 5.6 Immunofluorescence assay

Following the behavioral tests, mice were anesthetized and perfused intracardially with 4% paraformaldehyde followed by normal saline, and their brains were extracted. Brain tissues were dehydrated in sucrose solutions, embedded in Optimal Cutting Temperature (OCT) compound (Tissue‐Tek), and sliced into 30‐µm‐thick sections using a cryostat (Leica). For immunofluorescence staining, brain sections were treated with phosphate‐buffered saline(PBS), permeabilized with Triton X‐100, blocked with bovine serum albumin (BSA), and incubated with primary antibody overnight at 4°C. The next day, sections were washed, incubated with Alexa Fluor 488‐conjugated secondary antibody, counterstained with 4′,6′‐diamidino‐2‐phenylindole (DAPI), mounted on glass slides with glycerol, cover‐slipped, and examined using confocal microscopy.

### 5.7 Western blotting

After homogenization, the concentration of protein in brain tissue or N2a‐APP cells was determined. β‐Mercaptoethanol and bromophenol blue were then added to the samples, followed by boiling at 100°C for 10 min to denature the protein. The extracted protein (15 µg per sample) was separated on 8−15% sodium dodecyl sulfate–polyacrylamide gel electrophoresis (SDS‐PAGE), transferred to polyvinylidene difluoride (PVDF) membranes (Millipore), blocked with 5% BSA in Tris‐buffered saline (TBS) with 0.1% Tween 20 (TBST) for 1 h and incubated with the appropriate primary antibodies overnight at 4°C. After washing with TBST, the membranes were incubated with horseradish peroxidase (HRP) conjugated secondary antibody for 1 h at room temperature, and then immersed in enhanced chemiluminescence (ECL) solution at a 1:1 ratio, and exposed to ECL‐Hyperfilm (Amersham, Germany).

### 5.8 Nissl staining

Coronal brain sections (30 µm thick) were washed with PBS, mounted on gelatin‐coated slides, allowed to dry for 30 min, and then stained with cresyl violet for 5 min and heated in the oven. Subsequently, the sections were quickly differentiated in 95% ethanol for 2 min, washed in distilled water for 2 min, dehydrated twice in anhydrous ethanol (5 min each time), cleared in xylene for 10 min, and sealed with neutral balsam. Finally, the brain slices were observed and imaged using an optical microscope.

### 5.9 Golgi staining

The Golgi‐Cox solution contained 5% potassium dichromate, 5% mercury chloride, 5% potassium chromate, and double‐distilled water at a volume ratio of 5:5:4:10. The solution was kept in the dark for approximately 1 week. For staining, the mice were perfused with 0.9% normal saline, and the whole brains were harvested and incubated in the Golgi–Cox solution for 3 days in the dark. The brain tissues were then transferred to a 1% silver nitrate solution and changed daily, soaked in darkness, and changed every 3 days. The brains were removed from the solution after 7 days and cut into 90‐µm‐thick sections using a vibrating microtome (Leica, Wetzlar, Germany).

### 5.10 Cell culture and treatment

Mouse neuroblastoma N2a cells with stable expression of APP (N2a‐APP) were cultured in DMEM (Keygen, Nanjing, China) containing 10% fetal bovine serum (Gibco, USA). N2a‐APP cells were seeded at a density of 1×10^6^ cells per well in 6‐cm plates. Twenty‐four hours postseeding, cells were treated with 5 µM SGK1 inhibitor GSK‐650394 (MedChemExpress, China) for 48 h. As the inhibitors were dissolved in dimethylsulfoxide (DMSO), the DMSO‐treated N2a‐APP cells were used as a control.

### 5.11 ELISA quantification of Aβ40 and Aβ42

Aβ40 and Aβ42 levels were quantified using the Human Aβ1‐40 or Aβ1‐42 ELISA Kit (Elabscience, China). Briefly, cell suspensions were homogenized in buffer (PBS with protease inhibitors), centrifuged at 2000×*g* for 25 min, and the supernatant added to anti‐Aβ‐coated micro ELISA plates. Following a 90‐min incubation, a biotinylated detection antibody was applied and incubated at 37°C for 1 h. After washing, HRP conjugate solution was added, followed by a 30‐min incubation. Subsequently, a substrate reagent was added and incubated for 15 min at 37°C. Optical density at 450 nm was measured using a microplate reader after adding the stop solution.

### 5.12 RNA extraction and detection

After dissecting the mouse brain, the brain tissue was quickly placed into a pre‐labeled 1.5‐milliliter EP tube and then rapidly frozen into tissue blocks in a cooling box containing liquid nitrogen to prevent RNA degradation. Subsequently, the EP tube containing the frozen tissue was placed on dry ice and transported to Novogene for RNA extraction using standard methods from the tissue. The extracted RNA samples underwent strict quality control, primarily assessed through the Agilent 2100 bioanalyzer to accurately detect RNA integrity before library preparation for sequencing.

### 5.13 Bioinformatics analysis

The data analysis and visualization were performed using the NovoMagic v3.0 platform (Beijing Nuohe Zhiyuan Technology Corp., Beijing, China). Differential gene volcano plots, KEGG pathway analysis, GSEA, GO enrichment analysis, and heat maps were generated through this platform. A PPI network was constructed using the STRING database version 11.5 (https://string‐db.org/).^37^ Signaling pathways were illustrated using Clue GO in Cytoscape software (version 3.8.2)^38^ to highlight DEGs. mRNA expression levels of ATP6V0D2 (from Hippocampus GSE36980) and CTSD (from Hippocampus GSE29378) were obtained from the AlzData database (http://www.alzdata.org/).^39^

### 5.14 Statistical analysis

All data are expressed as means ± standard error of the mean (SEM). Statistical significance was analyzed by one‐way analysis of variance (ANOVA), two‐way ANOVA, or unpaired Student's *t*‐tests. A *p*‐value < 0.05 was considered significant.

## AUTHOR CONTRIBUTIONS

Xiaochuan Wang designed all experiments and organized all results. Zhendong Xu planned and performed all experiments and participated in the writing of the manuscript. Jichang Hu, Zhen Wei, Henok Kessete Afewerky, Yang Gao, Lu Wan., Longfei Li, Ling Lei, Yi Liu, Fang Huang, Tong Yu, Jian‐Zhi Wang, Hong‐Lian Li, and Rong Liu analyzed the data. Yu Lei analyzed and interpreted the data. All authors have read and approved the final manuscript.

## CONFLICT OF INTEREST STATEMENT

The authors declare that they have no competing interests.

## ETHICS APPROVAL AND CONSENT TO PARTICIPATE

The study protocol was approved by the Ethics Committee of Huazhong University of Science and Technology (Wuhan, Hubei, China; IACUC number: 3308).

## Supporting information

Supporting information

## Data Availability

The datasets used and/or analyzed are available from the corresponding author upon reasonable request.
