# Supplementary material for: Dynamic changes in lysosome‐related pathways in APP/PS1 mice with aging
Source: MedComm (2020). 2024 Apr 10;5(4):e540. doi: 10.1002/mco2.540 (PMC11006716; doi:10.1002/mco2.540)
Supplement: Supplementary file 1 — Supporting information [file MCO2-5-e540-s001.docx]

**Temporal Changes in Lysosome-related Pathways in APP/PS1 Mice with Aging**

- **Dynamic change of degradation system in AD**

Zhendong Xu^1^, Jichang Hu^1^, Zhen Wei^1^, Yu Lei^1^, Henok Kessete Afewerky^1^, Yang Gao^1^, Lu Wan^1^, Longfei Li^1^, Ling Lei^1^, Yi Liu^1^, Fang Huang^1^, Tong Yu^1^, Jian-Zhi Wang^1,2^, Hong-Lian Li^1^, Rong Liu^1^, Xiaochuan Wang^1,2†^

^1^Department of Pathophysiology, School of Basic Medicine, Key Laboratory of Education Ministry/Hubei Province of China for Neurological Disorders, Tongji Medical College, Huazhong University of Science and Technology, Wuhan 430030, China

^2^Co-innovation Center of Neuroregeneration, Nantong University, Nantong, JS 226001, China

**† Correspondence to:**

Xiaochuan Wang, Ph.D., Professor, Department of Pathophysiology, School of Basic Medicine, Tongji Medical College, Huazhong University of Science and Technology, Wuhan 430030, China.

Email: wangxiaochuan@hust.edu.cn

**Acknowledgments**

We are grateful to Prof. Yueguang Rong (Department of Pathogen Biology, School of Basic Medicine, Tongji Medical College, Huazhong University of Science and Technology, Wuhan, China) for helpful and constructive technical suggestions during the research process, and to the Medical Subcenter of HUST Analytical & Testing Center for data acquisition.

**Supplementary Figure legends**

**Supplementary Figure 1**. Age-dependent anxious behavior in APP/PS1 mice. APP/PS1 mice aged 5 months, 9 months and 13 months were divided into three groups according to age gradient, and c57 wild-type mice aged 5 months were used as controls, and then behavioral tests were conducted. (A-C) Escape latency of MWM. (D-G) Open field test and Elevated plus maze test were performed to assess the anxiety-like behavior of mice. (D) Representative trajectory. (E) Center duration. (F) Open-Arm entry. (G) Open-Arm duration. Data are presented as Mean ± SEM (5M Ctr, n= 10; 5M APP/PS1, n= 10; 9M Ctr, n= 10; 9M APP/PS1, n= 10; 13M Ctr, n= 9; 13M APP/PS1, n= 8, P value significance is calculated from a one-way ANOVA with Tukey multiple comparison test. ns: no significant).

**Supplementary Figure 2**. There is no deposition of aβ-amyloid plaque in the cortex and hippocampus of age-gradient c57 mice. (A) Levels of APP in the hippocampus were detected by westernblot analysis, and β-actin was used as a loading control. (B) Quantitative analysis of the blots. (C) Representative immunofluorescence staining images showing amyloid-β (Aβ) deposits in the hippocampus and cortex of age-gradient c57 mice. (D) The Venn diagram to find genes shared between 5 month-old (AH5) APP/PS1 mice versus 5 month-old (CH5) WT mice, 9 month-old (AH9) APP/PS1 mice versus 9-month-old (CH9) WT mice and 13-month-old (AH13) APP/PS1 mice versus 13-month-old (CH13) WT mice. Data are presented as Mean ± SEM (n=3 per group *P<0.05, **P<0.01, ***P<0.001, ****P<0.0001).

**Supplementary Figure 3.** The difference genes and enrichment pathways of age gradient APP/PS1 mice are significantly different from that of age gradient c57 mice. (A-C) The different expression genes of the hippocampus between APP/PS1 mice and c57 mice. (D-F) Top20 KEGG pathways most enriched in the different gene expressions of the hippocampus between APP/PS1 mice and c57 mice.

**Supplementary Figure 4**. Comparison of the expression differences in ATP6V0D2 and CTSD transcription levels between Alzheimer's disease patients and healthy individuals. (A-B) The mRNA expression level of ATP6V0D2 (Hippocampus GSE36980) and CTSD (Hippocampus GSE29378) derived from AlzData database (http://www.alzdata.org/).

**Supplementary Figure 5**. The lysosomal function had no significant change in the c57 mice hippocampus with increased age. (A) Western blotting was performed to detect the expression of ATP6V0D2 and CTSD proteins in the hippocampus. (B-C) Quantitative analysis of the blots. Data are presented as Mean ± SEM (n=3 per group *P<0.05, **P<0.01, ***P<0.001). (D) Gene Ontology (GO) analysis of the differential genes (in Figure 3D) using Clue GO software and the p-value was set at <0.05. (E) The heatmap of differential expression genes in the lysosome pathway of six groups of mice. (F) Schematic drawing of the Lysosome pathway (mmu04142). Red font indicates the location of target genes (Atp6v0d2 and Ctsd) involved in the Lysosome pathway pathway.

**Supplementary Figure 6.** The endosome, autophagy and FOXO3a pathways had no significant change in c57 mice hippocampus as age increased. (A) Western blotting was performed to detect the protein in the endosome, autophagy and FOXO3a pathways. (B-C) Quantitative analysis of the blots about the endosome-lysosome pathway. (D-E) Quantitative analysis of the blots about the autophagy-lysosome pathway. (F) Quantitative analysis of the blots about the FOXO3a pathway. Data are presented as Mean ± SEM (n=3 per group *P<0.05, **P<0.01, ***P<0.001).

**Supplementary Figure 7.** Discovery of SGK1/FOXO3a pathway alteration in the hippocampus of APP/PS1 mice using bioinformatics methods. (A)The Schematic drawing of differential expression gene in SGK1/FOXO3a pathway of four groups of mice using Clue GO software and the p-value was set at <0.05. (B) The heatmap of differential expression genes in the SGK1/FOXO3a pathway of four groups of mice. (C) PPI network of targets extracted from (B). (D) The Venn diagram to find genes shared between 13-month-old (AH13) APP/PS1 mice versus 5-months-old (AH5) APP/PS1 mice and 13-month-old (AH13) APP/PS1 mice versus 13-month-old (CH13) WT mice. (E) KEGG pathway analysis of the genes (Foxo3 and Sgk1) using Clue GO software and the p-value was set at <0.05.

**Supplementary Figure 8.** Treatment with SGK1 inhibitor GSK-650394 in AD model cell N2a-APP resulted in abnormal metabolism of APP and increased Aβ toxicity. (A) Western blotting was performed to detect the protein in APP metabolism and FOXO3a pathways. (B) Quantitative analysis of the blots about the FOXO3a pathway. (C-D) Quantitative analysis of the blots about the levels of APP and sAPPβ. (E-G) Quantitative analysis of the blots about the cleaving enzymes in APP metabolism. (H-I) The AD model cell N2a-APP homogenates were assayed for Aβ1-40 (H) or Aβ1-42 (I) levels. Data are presented as Mean ± SEM (n=3 per group, P value significance is calculated from Student's t tests. ns: no significant, *P<0.05, **P<0.01, ***P<0.001).

**Supplementary Figure 1**


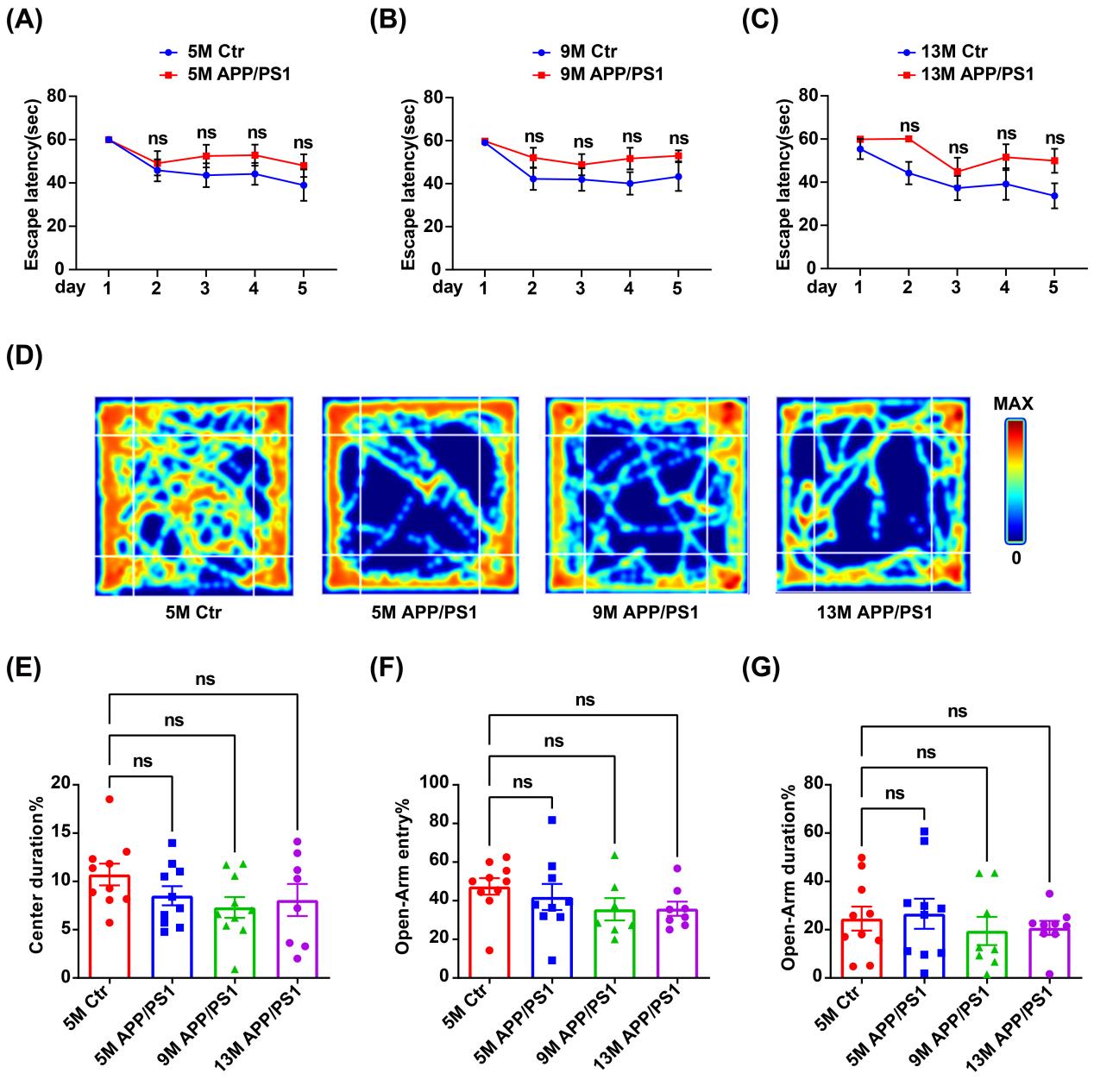


**Supplementary Figure 2**


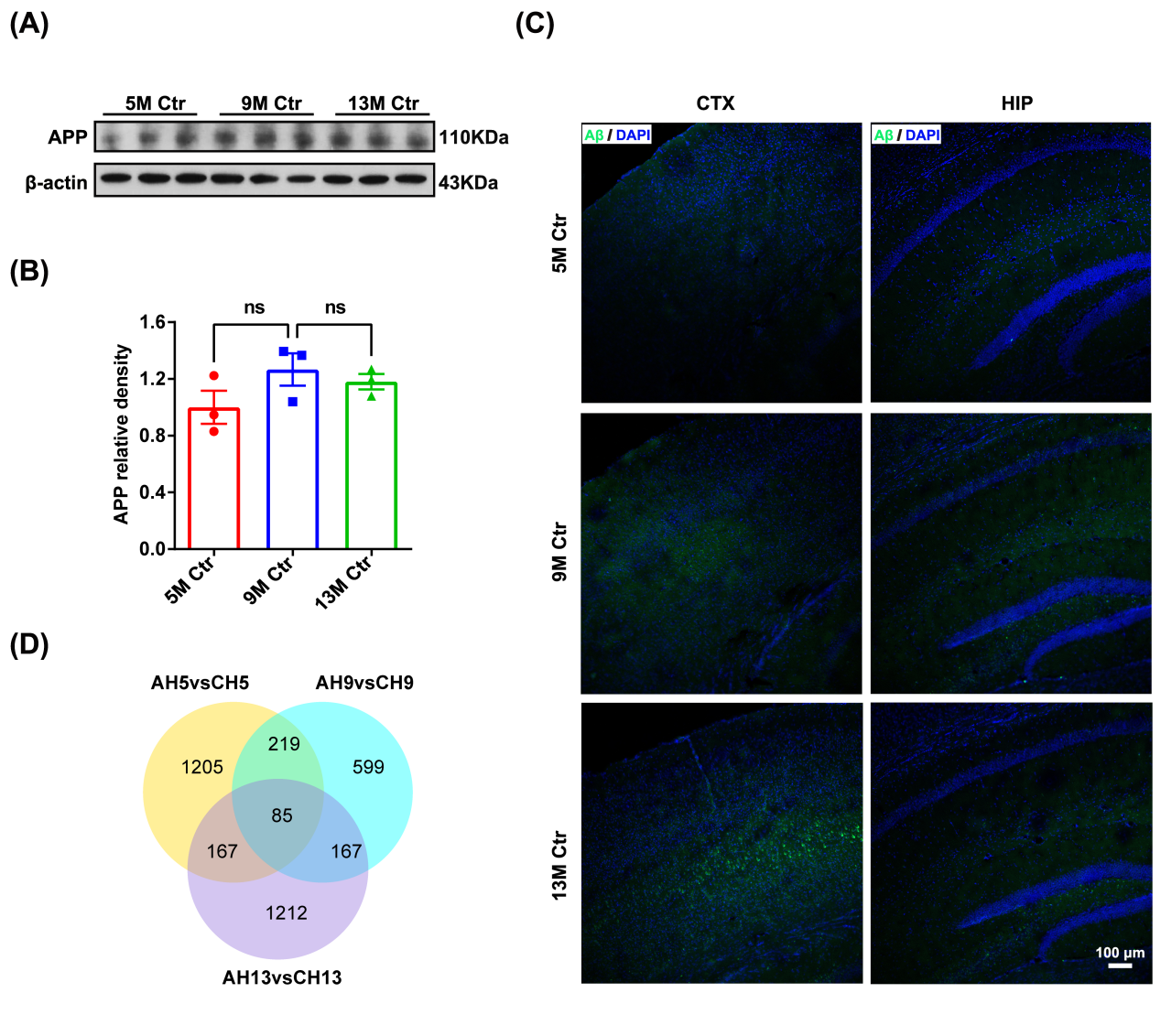


**Supplementary Figure 3**



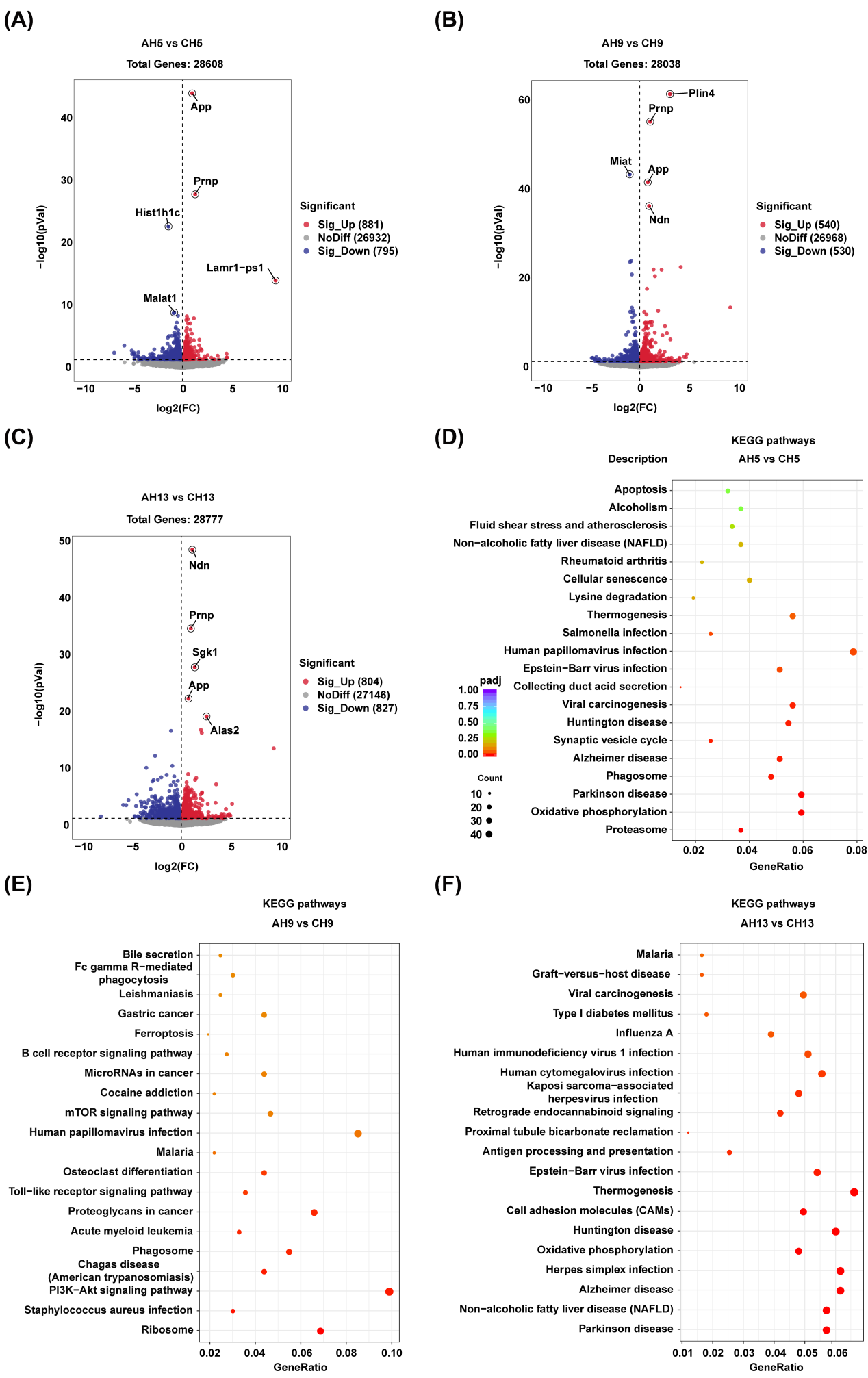


**Supplementary Figure 4**

**
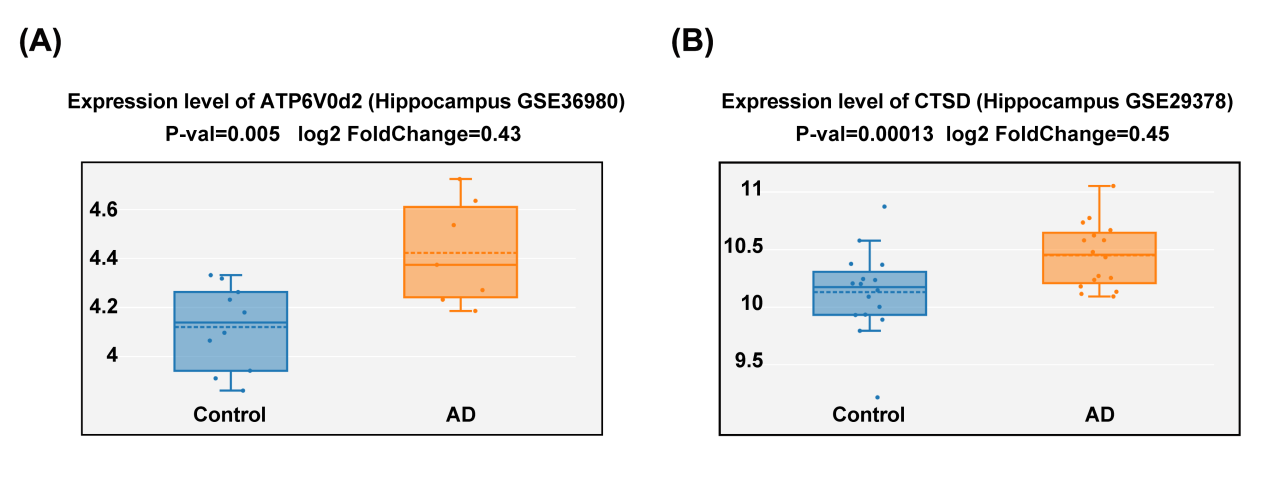
**

**Supplementary Figure 5**


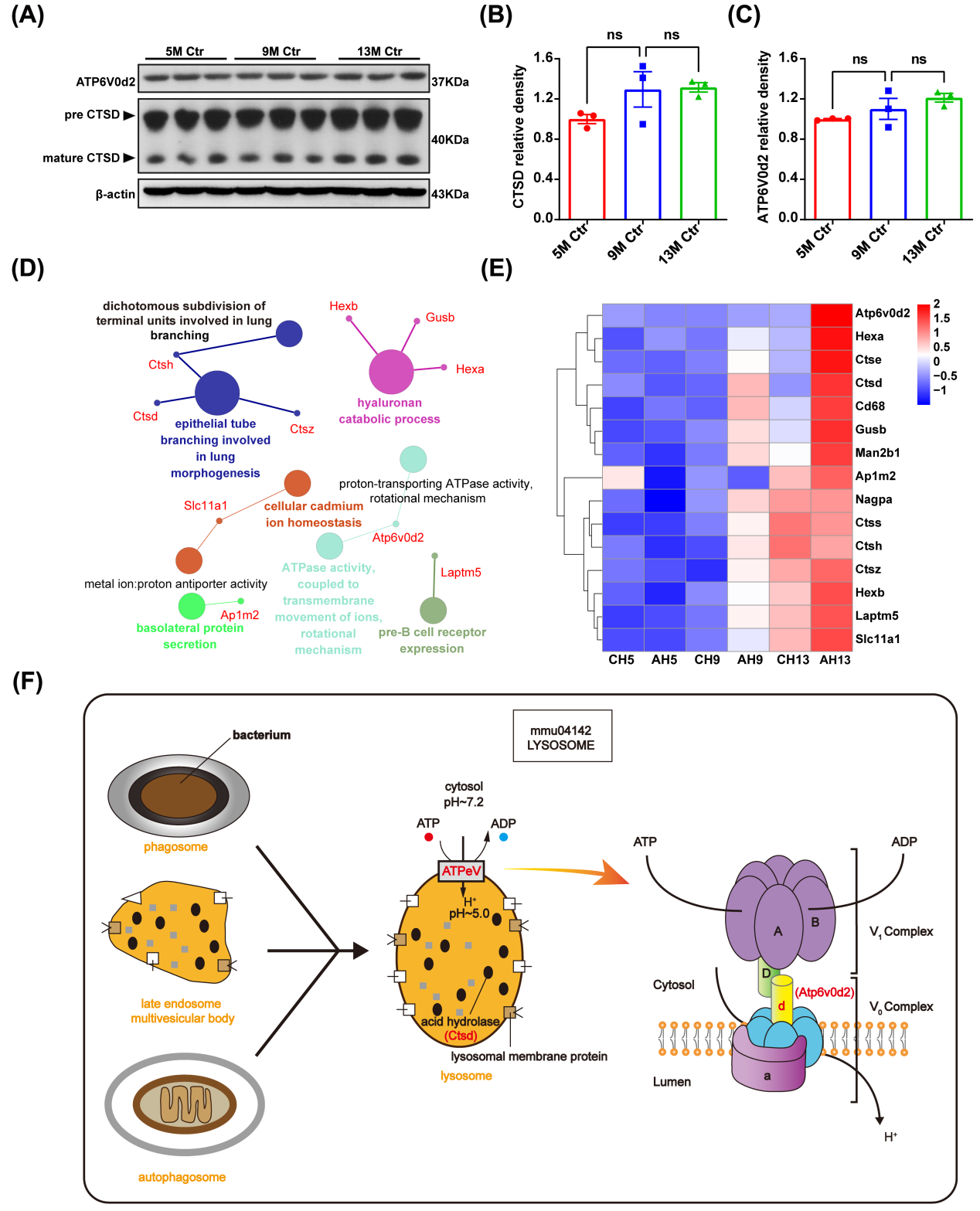


**Supplementary Figure 6**


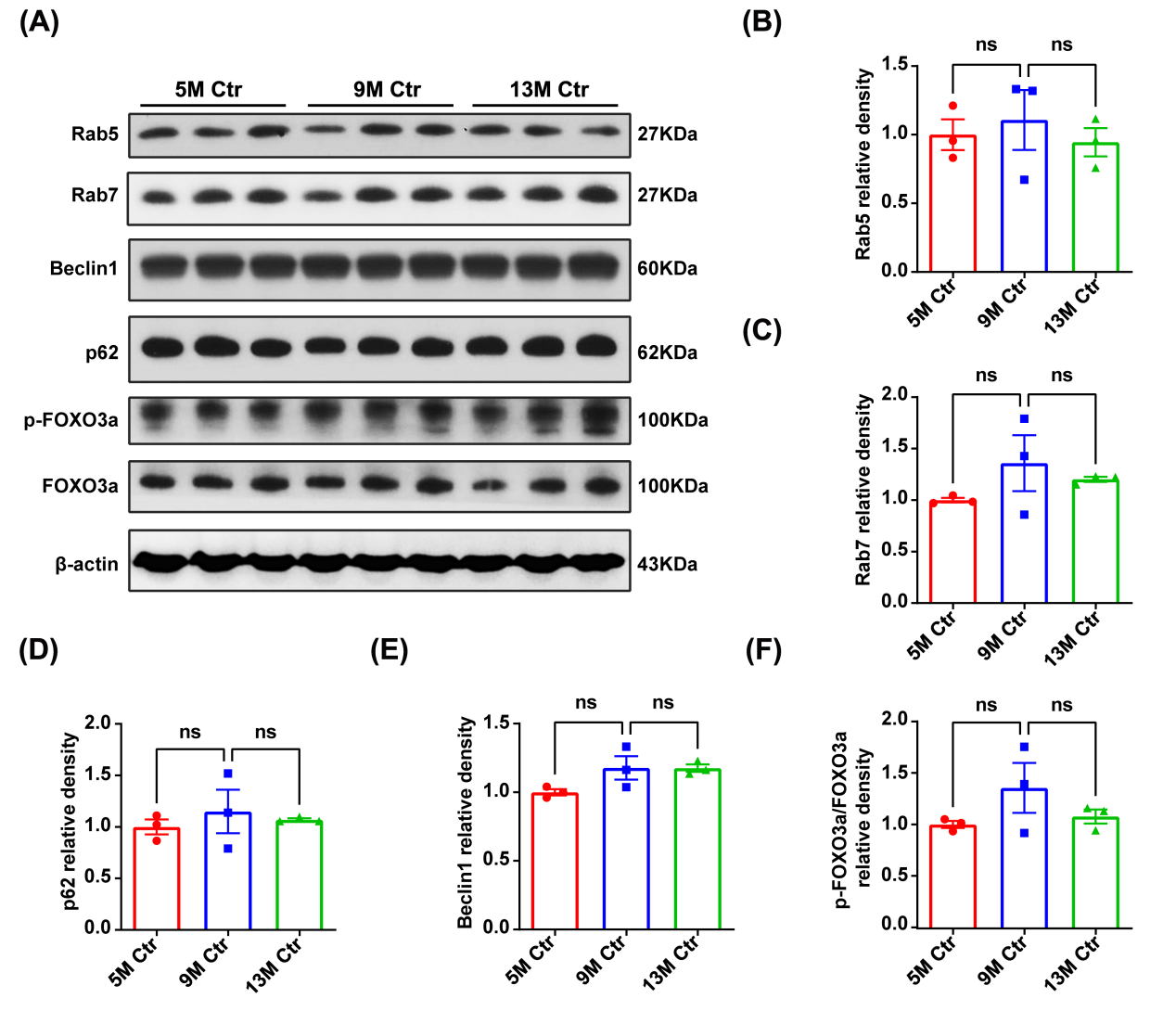


**Supplementary Figure 7**

**
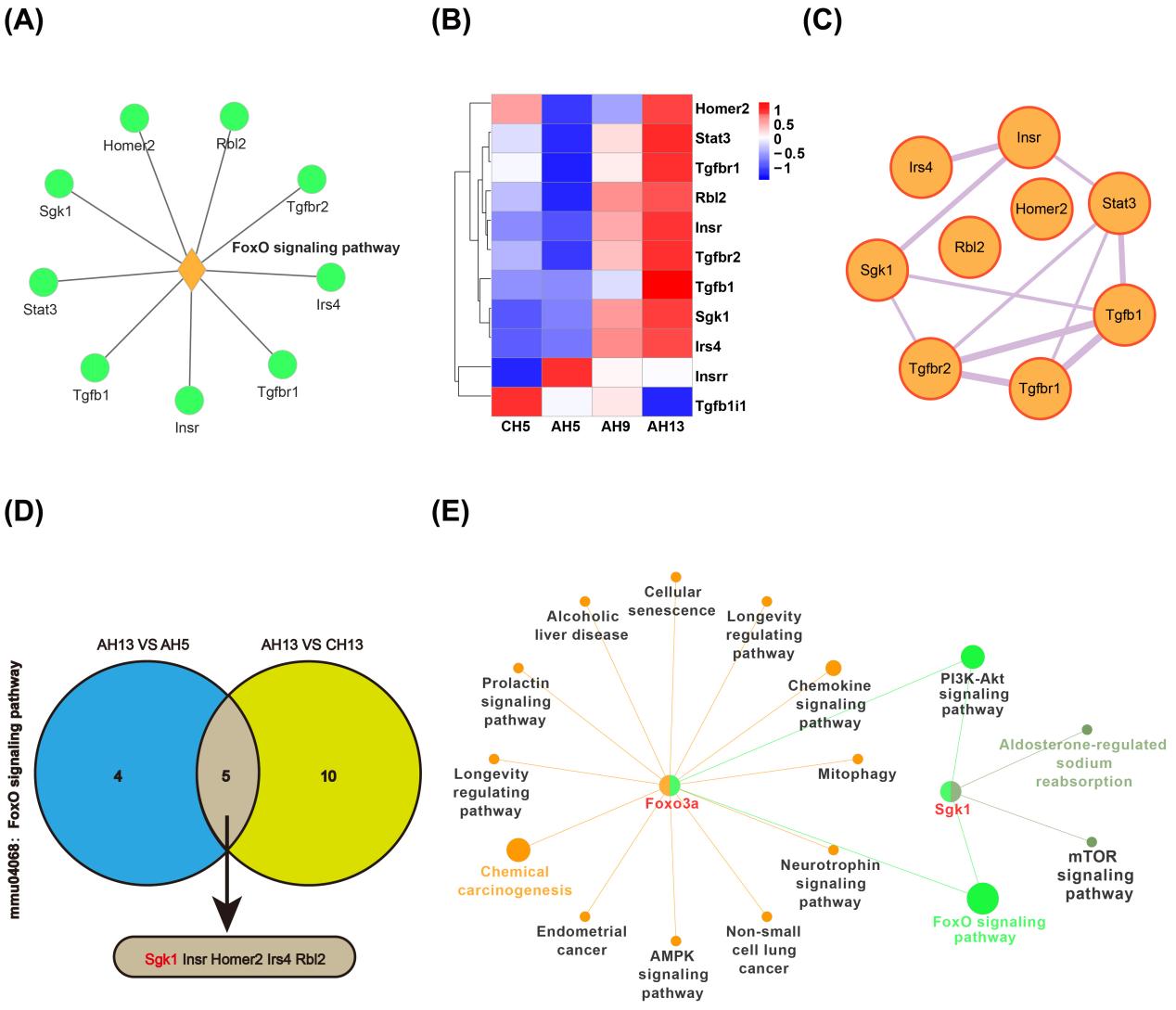
**

**Supplementary Figure 8**


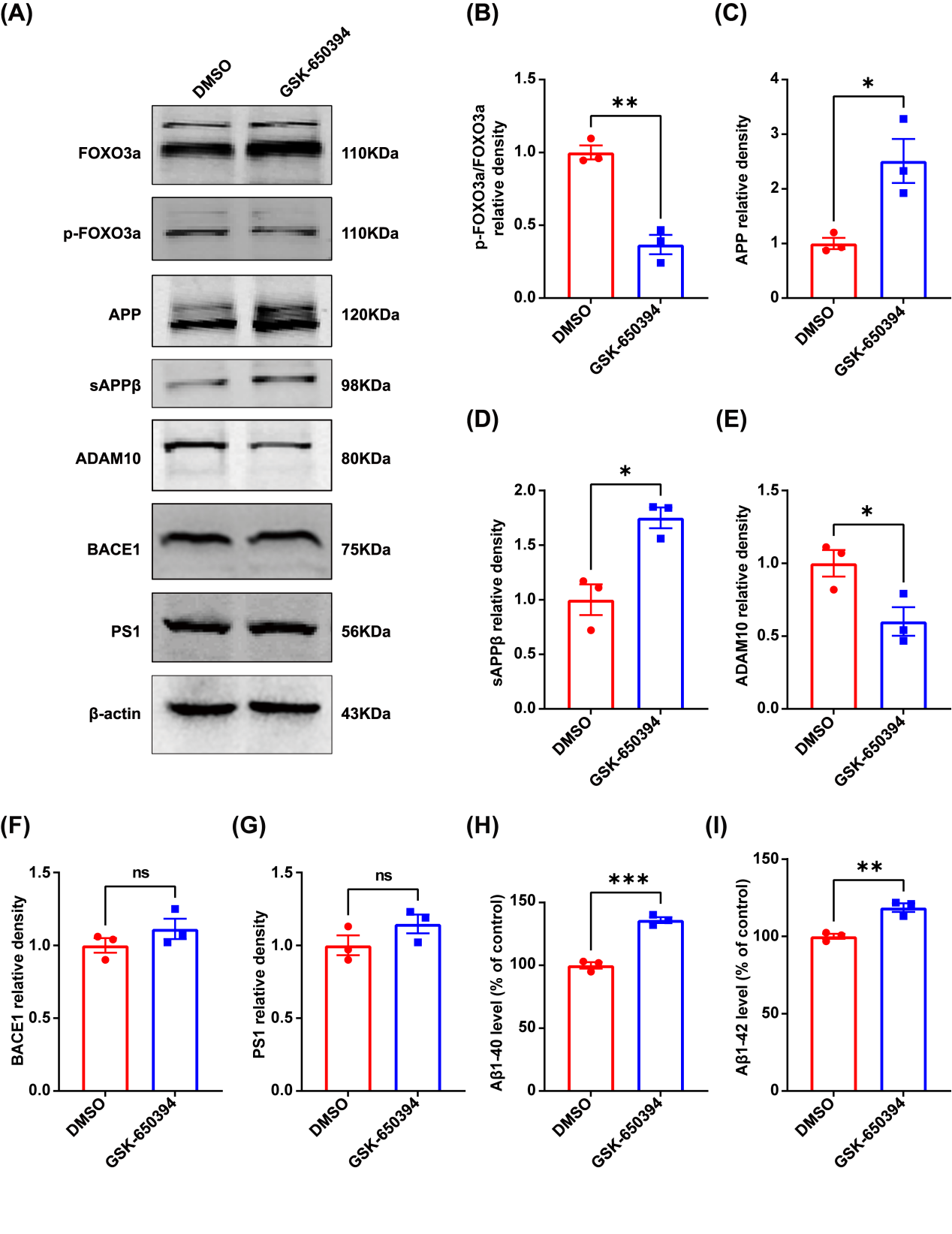


**Table S1. Antibodies employed in this study**

| Antibody | Reactivity | Dilution | | Application | Company | |
| --- | --- | --- | --- | --- | --- | --- |
| APP | Rabit | 1:1000 | WB | | | Proteintech |
| CTSD | Rabit | 1:1000 | WB | | | CST |
| ATP6V0d2 | Mouse | 1:1000 | WB | | | Abcam |
| Rab5 | Rabit | 1:1000 | WB | | | CST |
| Rab7 | Rabit | 1:1000 | WB | | | CST |
| LC3-Ⅱ | Rabit | 1:1000 | WB | | | Sigma |
| p62 | Mouse | 1:1000 | WB | | | Abcam |
| mTOR | Rabit | 1:1000 | WB | | | CST |
| P-mTOR | Rabit | 1:1000 | WB | | | CST |
| Beclin1 | Rabit | 1:1000 | WB | | | CST |
| GAPDH | Mouse | 1:1000 | WB | | | Beyotime |
| β-actin | Rabit | 1:1000 | WB | | | Abclonal |
| SGK1 | Rabit | 1:1000 | WB | | | Abcam |
| FOXO3a | Rabit | 1:1000 | WB | | | CST |
| P-FOXO3a | Rabit | 1:1000 | WB | | | CST |
| Aβ (6E10) | Mouse | 1:200 | IF | | | Biolegend |
| ADAM10 | Rabit | 1:1000 | WB | | | Zenbio |
| sAPPβ | Rabit | 1:1000 | WB | | | IBL |
| BACE1 | Rabit | 1:1000 | WB | | | Zenbio |
| PS1 | Rabit | 1:1000 | WB | | | Sigma |

WB, western blot; IF, immunofluorescence.
